# Rethinking Health Systems Responsiveness in Low- and Middle-Income Countries: Validation Study

**DOI:** 10.2196/59836

**Published:** 2024-09-18

**Authors:** Meesha Iqbal, Cecilia Ganduglia Cazaban, Robert Morgan, Cici Bauer, Sameen Siddiqi

**Affiliations:** 1 School of Public Health, the University of Texas Health Science Center at Houston Houston, TX United States; 2 Aga Khan University Karachi Pakistan

**Keywords:** health system, healthcare system, health care, responsiveness, Pakistan, Low-and middle-income countries

## Abstract

**Background:**

Health systems responsiveness (HSR) is the ability of systems to respond to legitimate non-health expectations of the population. The concept of HSR by the World Health Organization (WHO) includes respect for dignity, individual autonomy, confidentiality, prompt attention to care, availability of basic amenities, choice of provider, access to social support networks, and clarity of communication. The WHO tool is applied globally to assess HSR in low, middle, and high-income countries.

**Objective:**

We have revised the conceptual framework of HSR following a rigorous systematic review and made it specific for low- and middle-income countries (L&MICs). This study is designed to (1) run the Delphi technique to validate the upgraded conceptual framework of HSR, (2) modify and upgrade the WHO measurement tool for assessing HSR in the context of L&MICs, and (3) determine the validity of the upgraded HSR measurement tool by pilot testing it in Pakistan.

**Methods:**

The Delphi technique will be run by inviting global public health experts to provide suggestions on the domains and subdomains of HSR specific to L&MICs. Cronbach ɑ will be calculated to determine internal consistency among the participants. The upgraded HSR conceptual framework will serve as a beacon to modify the measurement tool by the research team, which will be reviewed by subject experts for refinement. The modified tool will be pilot-tested by administering it to 1128 participants from primary, secondary, and tertiary care hospitals in Rawalpindi district, Pakistan. Additionally, an “observation checklist” of HSR domains and subdomains will be completed to objectively measure the state of HSR across health care facilities. HSR assessment will be further strengthened by incorporating the perspective of hospital managers, service providers, and policy makers (ie, the supply side) as well as community leaders and representatives (ie, the demand side) through qualitative interviews.

**Results:**

The study was started in January 2024 and will continue until February 2025. A multidimensional approach will yield significant quantifiable information on HSR from the demand and supply sides of L&MICs.

**Conclusions:**

This study will provide a conceptual understanding of HSR and a corresponding measurement tool specific to L&MICs. It will contribute to global public health literature and provide a snapshot of HSR in Rawalpindi district, Pakistan, with concrete action points for policy makers.

**International Registered Report Identifier (IRRID):**

DERR1-10.2196/59836

## Introduction

Responsiveness to legitimate non-health expectations of the population is listed as one of the three intrinsic goals of health systems by the World Health Organization (WHO), alongside improving health and providing financial risk protection [1]. Health systems responsiveness (HSR) relates to a “system’s ability to respond to legitimate expectations of potential users about non-health enhancing aspects of care” [2]. The concept of HSR arose in the late 1990s [3] and was formalized in the landmark WHO report of 2000—“Health Systems: Improving Performance” [1]. Responsiveness is a social goal that emphasizes health systems should serve people, going beyond merely assessing people’s satisfaction with the medical care they receive [3]. The World Health Report [1] defined HSR based on 7 domains, including respect for dignity, autonomy, confidentiality, prompt attention, quality of basic amenities, access to social support networks (particularly for inpatients), and choice of provider [1]. An additional domain of “clarity of communication” was subsequently added [2,4,5].

The significance of HSR has been repeatedly emphasized in the literature. The concept of responsiveness has its origin in Donabedian’s framework of quality of care [6] and reflects the protection of human rights and dignity [4]. Improving HSR is essential to making progress toward achieving universal health coverage (Target 3.8 of Sustainable Development Goals) [7], which entails that everyone should receive good quality health services without incurring financial hardship [7]. HSR ensures a higher level of comfort for people [4] and is the most important predictor of client satisfaction rather than clinical competence [8-10]. It is suggested that aspects of HSR contribute to a patient’s willingness to return to health care facilities and continue engaging in health care [11]. It is also suggested that improving HSR expands service coverage [6,12-14], decreases noncompliance with treatment regimens [15-17], and raises the chances of treatment success [18]. Evidence from Nigeria demonstrates that people’s trust in the system improves the uptake of maternal health services among pregnant women [19,20]. From the perspective of policy makers, responsiveness can be improved with minimal investment in sophisticated technology, equipment, and human resources [21,22] compared to clinical aspects of health care. This has the dual impact of increasing client satisfaction and improving overall population health.

Although literature pertaining to HSR has been growing in the past 2 decades [23], conceptual clarity of its domains and subdomains has received little attention [19,24]. Ambiguity originates due to contextual variation, leading different researchers to address HSR in varied ways [25-30]. The terms “Health Systems’ Responsiveness*,*” “Health Service Responsiveness” and “Human Resource for Health (HRH) Responsiveness” have been used interchangeably and have generally referred to the user-service interface of patients and health care providers rather than addressing health systems responsiveness at a systemic level [19,24]. It is not evident what comprises a response, what the legitimate expectations of people are, at what level a response is anticipated (provider or systemic), and for whom the response is meant (patients, individuals, or the general public).

Varied understandings and interpretations of HSR have led to gaps in its scientific assessment as well as strategies to improve it. The most common measurement tool to assess HSR is the WHO tool utilized in the Multi-Country Survey study (2000-2001, across 61 countries) [31] and the World Health Survey (2002-2004, across 71 countries) [32]. It has been universally applied across high, middle, and low-income countries. The WHO HSR conceptual framework and measurement tool have received technical criticism from researchers around the globe. It is highlighted that the 8 domains of HSR skip important concepts, including but not limited to the quality of counseling, follow-up care, trust, coordination of the course of treatment, the quantity of information provided, and language barriers [33-35]. The most cited drawback of the WHO measurement tool is its universal application to countries of all income levels, with drastically varied social, cultural, and economic contexts. The health and non-health expectations of the populations of high-income countries as well as low- and middle-income countries (L&MICs) also differ, as these are embedded in the broader sociocultural macrosystem. It is stated that domains such as accountability of errors, ease of scheduling appointments, coverage during known provider absences, shared sense of urgency, and expression of empathy are critical for people in high-income countries, whereas these domains are not significant to people in L&MICs [36]. It is crucial to assess HSR in L&MICs, which generally have limited capital to invest in high-cost technology for improving clinical care. Investing in HSR is cost effective and can help countries make progress toward achieving universal health coverage as part of their commitment toward Sustainable Development Goals [7].

We designed this study to address the criticism and limitations of the WHO HSR conceptual framework and measurement tool. This study will revise and validate the concept of HSR (conceptual framework) as presented by the WHO 2000 report [1] and provide a comprehensive tool for its measurement, which is specific to the context of L&MICs. The specific objectives of the study are to (1) validate the upgraded conceptual framework of HSR (explained in the *Methods* section) by applying the Delphi process; (2) to revise the WHO measurement tool for assessing HSR; and (3) to validate the upgraded tool by pilot-testing it in primary, secondary, and tertiary care hospitals of Rawalpindi district, Pakistan.

## Methods

### Preliminary Work: Modification of the HSR Conceptual Framework and Measurement Tool

The WHO HSR framework was developed more than 2 decades ago through a literature review, examining surveys related to patient satisfaction and discussions with public health professionals and researchers involved in the health sector [5]. We have taken a similar approach to refine the HSR framework: a literature review followed by suggestions for modifications by global public health experts.

**Figure 1:**
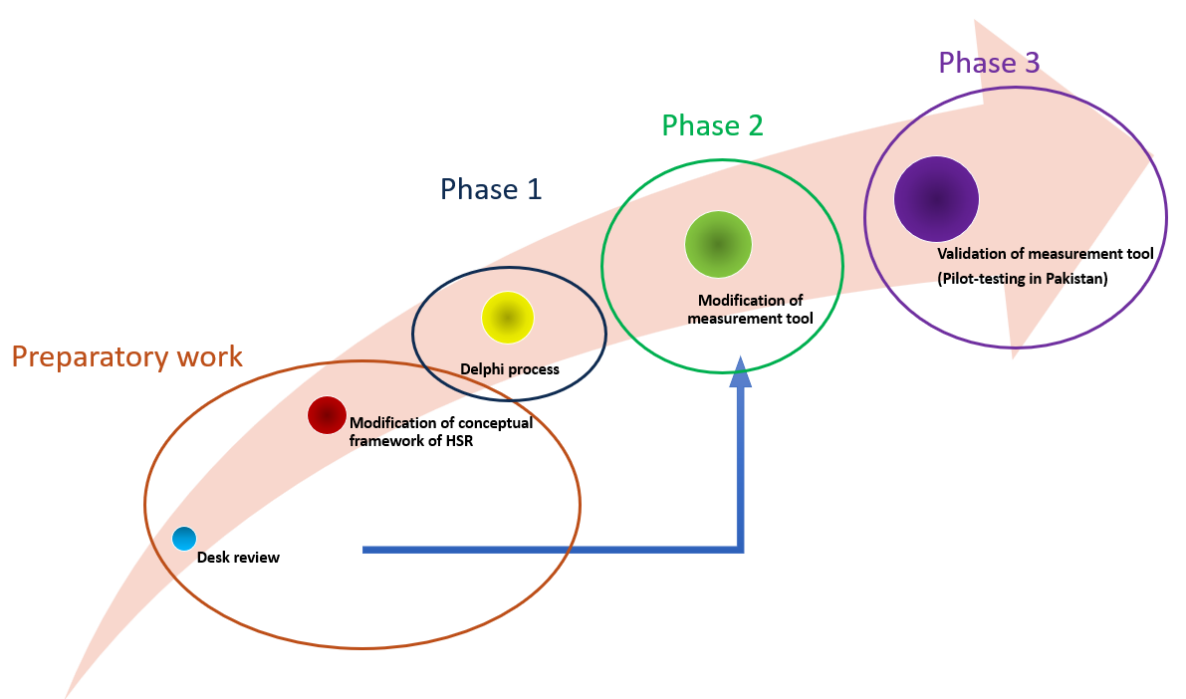
Phases of the study.

A systematic review was carried out by the research team in 2023 to revise and upgrade, (1) the WHO conceptual framework and (2) the measurement tool of HSR to suit the context of L&MICs. A search was carried out using the PubMed and Google Scholar databases, focusing on constructs related to “health systems” (Medical Subject Headings terms) and “responsiveness.” The initial number of hits was 844 records, and 19 articles were included after screening [37-54]. These articles were specifically related to HSR in L&MICs and addressed the HSR conceptual framework and/or the measurement tool. The details of the systematic review and retrieval of literature are provided in Multimedia Appendix 1. Table 1 elaborates on the modified version of the HSR conceptual framework, based on the systematic review. We have added the following 4 new domains: building trust, guidance, financial sensitivity, and coordination and continuity of care. The existing domain of “prompt attention” has been changed to “access to care.” Clarity of communication” has been modified to “attention and clarity of communication.” The WHO measurement tool has been revised by the research team in concordance with the conceptual framework (Multimedia Appendix 2). Starting with the revised and upgraded HSR conceptual framework and measurement tool, we aim to validate both in the proposed study. The study will be conducted in concrete phases that align with each objective, as shown in Figure 1.

**Table 1:** The revised conceptual framework of health systems responsiveness (HSR), based on systematic review.

Domains of HSR	Definition/concept	Elements of the domain
1. Respect for dignity	At the extreme, this means not sterilizing individuals with a genetic disorder or locking up people with communicable diseases, which would violate basic human rights. More generally, it means not humiliating or demeaning patients.	Treatment with respect by health care staff. Patients should be welcomed at the health care unit, addressed respectfully at all times, and not shouted at or abused. Privacy during examination and treatment. Safeguarding of human rights (eg, freedom of movement for patients of leprosy or tuberculosis).
2. Respect for autonomy	This refers to freedom of patients to participate in choices about their own health. It includes helping choose what treatment to receive or not to receive.	Right of an individual to information about their disease and alternative treatment options. Right to be consulted about treatment. *Informed consent in the context of testing and treatment*. The right of patients of sound mind to refuse treatment.
3. Respect for confidentiality	It refers to the right to determine who has access to one’s personal health-related information.	Conducting consultations with the patients in a manner that protects their privacy. Safeguarding the confidentiality of information provided by the patient and information relating to an individual’s illness (except where such information needs to be given to another health care provider).
4. Quality of basic amenities	It refers to the quality of the environment in which health care is provided.	Clean water, clean toilets, and clean linen. Sufficient ventilation (fresh air). Clean surroundings (regular procedures for cleaning and maintenance of hospital buildings and premises). Healthy and edible food. Adequate furniture and seating.
5. Access to social support networks during care	It refers to provision of social needs for people receiving health care. It only applies to people receiving inpatient care.	Patients should be allowed visits by relatives and friends. Provision of food and other consumables by relatives and friends, if not provided by the hospital. Religious practices that do not interfere with hospital activities or offend others’ sensibilities. Access to newspapers, radio, and TV *Support to the family of patients (caregivers)* [22]. *Social financial networks*.
6. Choice of provider	It refers to the freedom to select which individual or organization can deliver one’s care.	Patients should be able to reach health services of choice without too much difficulty. Within a health care unit, individuals should be able to choose their health care provider. Individuals should be able to get a second opinion in cases of severe or chronic illness or surgery. Individuals should be able to get general and specialist care as appropriate *Choice of gender of the provider^b^* [31].
7. Prompt access to care^a^	Health care facilities should be geographically accessible–taking account of distance, transport, and terrain. People should also be promptly given care once in the health care setting.	Patients should be entitled to rapid care in emergencies. Patients should be entitled to care within reasonable time periods even in case of nonemergency health care problems. Waiting times for consultation and treatment should be reasonable.
8. Attention and clarity of communication^c^	Whether proper attention was given to the patient by health care providers and if there was appropriate communication.	Clarity in conveying information and evoking understanding. *Providing time for patients to understand their symptoms and to ask questions (Enough time)* [31]. *Insightful listening^b^* [32]. *No interruptions during consultation (unnecessary calls, texting, chatting, or singing)^b^* [28]. *Not using jargon^b^* [26]. *Asking patients if they understood the explanation (quality of counseling)* [32].
9. *Building trust*^*d*^ [26-28,35]	*The health care provider is advised to maximize the patient’s benefit, not tomaximize their own benefit.*	*Service oriented, not business-like behavior:* *Being asked to do tests from specified diagnostic centers.* *Visiting patients privately (by public sector physician)—moonlighting.* *Not being involved in illegal activities:* *Bringing patients in own private clinics.* *Accepting gifts from pharmaceutical representatives—prescribing substandard medicine.* *Mechanisms of accountability.* *Earning trust of patients.*
10. *Guidance*^*d*^ [22,26]	*General information on maintaining healthy lifestyle and specific guidance on the disease under consideration.*	*Information and suggestions on healthy lifestyle in general (eg, smoking cessation, physical activity, and healthy diet).* *Information and suggestions on disease prevention.* *Facilitating follow-up.* *Explaining access to medicines and diagnostic services* [37].
11. *Financial sensitivity*^*d*^ [26,28]	*It refers to how mindful health care providers are to the financial burden put on patients due to illness.*	*Trying to understand socioeconomic status of the patient.* *Considering socioeconomic status of the patient in discussing management options.* *Informing the cost of treatment/financial counseling.* *Providing financial assistance if needed—referring to organizations or individuals that can provide financial assistance.*
*12. Coordination and continuity of care^d^* [26,31,32,35]	*It refers to the extent to which care progresses smoothly as the patient moves across different health care providers and sectors.*	*Continuity of care (communication between providers).* *Referral services.*

^a^Previously, this domain was “prompt attention” in the World Health Organization (WHO) framework of responsiveness.

^b^New additions to the original WHO domains of responsiveness.

^c^“Attention” has been added to “clarity of communication*.”*

^d^Newly added domains. The italicized parts are new additions.

### Phase 1: the Delphi Technique

The revised conceptual framework (Table 1) will be run through the Delphi technique to validate its components (ie, domains and elements) and add robustness. All panel members included in the Delphi process will be experts in public health with a focus on health systems and/or HSR. We identified potential Delphi participants during literature review. Additional participants will be identified through a consultative process within the research team, supplemented by snowball sampling. They will be invited via email to participate in the study. Each participant will grade domains and elements of HSR according to “scientific strength” (face and content validity), “importance and relevance,” and “feasibility of measurement.” The grading will be recorded on a scale of 1 to 5, with 5 being the best and 1 being the worst score (1= strongly reject; 2= reject; 3= no opinion; 4= support; and 5= strongly support). They will also be requested to give percentage weight to each domain of HSR according to its importance. The domains and elements will be included in the conceptual framework if relevance and scientific strength are rated 4 or 5 by 70% or more of the experts and if feasibility is rated 4 or 5 by 50% or more of the experts [55].

### Phase 2: Modification of the WHO Measurement Tool

The research team has already modified the WHO responsiveness assessment tool in concordance with the upgraded conceptual framework (Multimedia Appendix 2). However, these edits are not final. The research team will revisit the tool after the Delphi process and make necessary edits. The tool will then be run via (1) expert review and (2) prepiloting the questions. We will contact 3 to 5 subject experts for expert review and request a remote meeting to discuss the measurement tool. All changes in the tool will be discussed with experts for their opinion. They will be asked for the relevance, importance, scientific strength, and feasibility of each question and to suggest question eliminations, modifications, and/or additions. Furthermore, appropriate response options (ie, Likert scale, binary scale, or open-ended responses) and tool analysis will be discussed. We intend to establish the content validity of the tool through this process. After the expert review, the principal investigator of the study will administer the tool to 7 to 10 adults aged >18 years from Rawalpindi district, Pakistan. These would not be subject experts. This will help determine whether the general public understands and comprehends the questions, as intended. Any necessary edits will be made to the tool based on their feedback.

### Phase 3: Pilot-Testing of the Measurement Tool and the HSR Assessment Methodology in Pakistan

**Figure 2:**
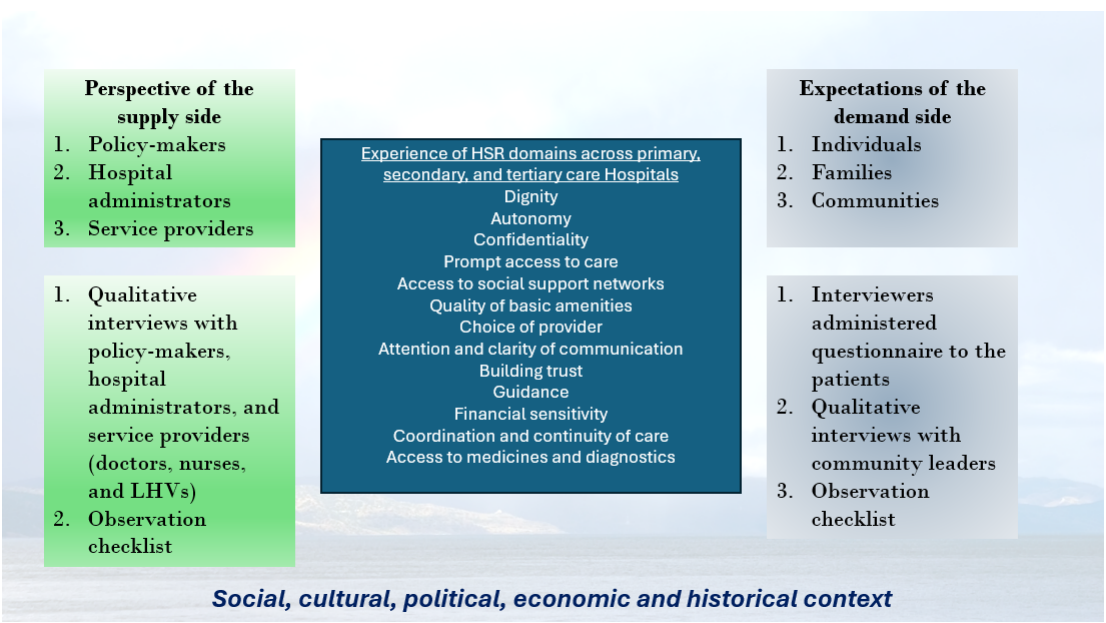
Analytical framework of the study (LHV: lady health visitor).

The tool will be pilot-tested in Rawalpindi district, Pakistan. Pakistan has been chosen for testing based on the feasibility of the research team. The survey will be based on three components: (1) facility-based survey, (2) community survey, and (3) key-informant survey**,** according to the analytical framework of the study (Figure 2; adapted and modified from Mirzoev et al [19]). The framework involves assessing HSR from the perspective of supply (ie, hospital managers, service providers, and policy makers) and demand (ie, patients and community members) sides in the broader social, cultural, political, economic, and historical context. A mixed methods approach will be adapted according to the framework.

#### Facility-Based Survey

**Figure 3:**
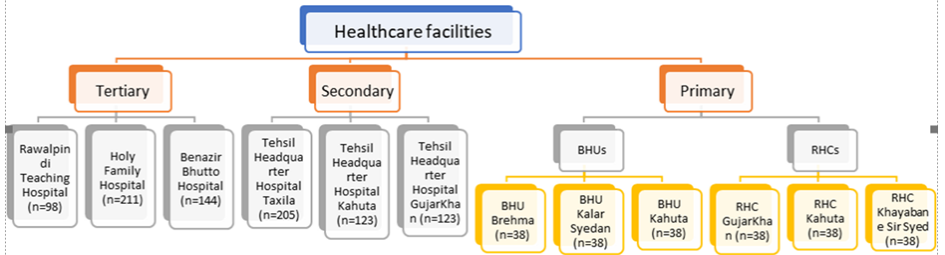
Sampling distribution across primary, secondary, and tertiary care hospitals (BHU: basic health unit; RHC: rural health center).

The facility-based survey will be a mixed methods study that will collect quantitative data from patients, qualitative data from hospital managers and service providers, and an observation checklist filled in by the research team. The semistructured interview guides and observation checklist are provided in Multimedia Appendix 3. The HSR measurement tool will be administered at the health care facilities. Participants will be sampled from three tiers of public health care facilities of Rawalpindi district, Pakistan. Data will be collected from 1128 participants spread across primary (basic health care units and rural health centers), secondary (Tehsil headquarters hospitals), and tertiary health care facilities. Figure 3 demonstrates the sample size distribution across the health care facilities of the Rawalpindi district. Multimedia Appendix 4 elaborates on sample size calculation and distribution as well as sampling strategy. The sample will be drawn from inpatients and outpatients across the departments of Medicine, Surgery, Gynecology/Obstetrics, and Pediatrics in secondary and tertiary care facilities according to probability proportionate to size sampling technique. Only outpatients will be included from primary care facilities. Purposive sampling will be done to recruit participants. Data will be collected from patients regarding their own or their child’s experiences of HSR. Any person aged ≥18 years who receives treatment from the health care facility the same day for themselves or their children (aged <12 years), does not have a reported mental illness, was not unconscious in the past 24 hours, understands and speaks Urdu, and is willing to participate in the study will be recruited as outpatients. Inpatient participants must have been hospitalized for at least 1 night in the past week at the hospital under study. Data will be electronically recorded using KoBo ToolKit. The distribution of all variables will be assessed, and outliers will be identified and assessed individually, case by case. Cases with extreme outlier measures will be eliminated from the dataset. Any variable with more than 50% missing information will be considered defective and removed from the data set. For the rest of the variables, missing values will be imputed by the “hot deck” method. Basic descriptive statistics will be calculated. Principal component analysis will be applied to the domains of HSR to generate a composite score of HSR according to the weights given to each category. The detailed analysis of the measurement tool will be discussed and finalized with subject experts (phase 2).

Hospital managers, general practitioners, specialists, nurses, and paramedic staff who have been working at the health care facility for the past year and are willing to participate in the study will be invited for qualitative interviews. A minimum of 3 interviews per secondary (n=9) and tertiary (n=9) health care facility and 1 within each primary health care facility (n=6) will be conducted (N=24). The research team will objectively determine the responsiveness of each health care facility using an observation checklist (total number of health care facilities =12) to minimize information bias (Multimedia Appendix 3).

#### Community-Based Survey

The research team will reach out to community representatives of Rawalpindi district to conduct in-person qualitative interviews and include their voices in the HSR assessment. They will be identified with the help of service providers, typically those working in primary health care facilities, through snowball sampling. A minimum of 15 community representatives will be included, and more will be added until the point of saturation is reached.

#### Key-Informant Survey

Key informant interviews will be carried out to include in-depth insights of policy makers in the study and understand the challenges, barriers, threats, opportunities, and possible solutions to improve HSR in Pakistan. We intend to apply the snowball sampling technique to interview 20 to 40 decision makers involved in the health sector in Pakistan. An effort will be made to include key people from the public and private sectors, academia, and WHO. As the country follows a devolved system, we also intend to capture representation from the federal government and the four provinces of Punjab, Sindh, KPK, and Balochistan.

All qualitative data will be transcribed and later analyzed using the directed content analysis technique [56], based on the conceptual framework of HSR. We will use Maxqda.v.24 for qualitative analysis. Codes, subthemes, and themes will be generated and organized in the broader umbrella of the HSR domains and elements. Word clouds and code matrices will be formed. Most qualitative information will be presented in narrative form, with verbatim excerpts supporting the quantitative information. Findings from all surveys will be triangulated and synthesized to generate meaningful information. Results will wholistically be displayed from the perspective of the demand and supply sides, according to the analytical framework of the study. The findings will help us formulate guiding principles and a strategic direction to improve HSR, culminating in potential action points for policy makers to help improve HSR and the overall health of the population.

### Ethical Considerations

Written informed consent will be obtained via email from participants in the Delphi technique. Verbal consent will be obtained and audio-recorded from participants in qualitative interviews (policy makers, hospital managers, and service providers). Written informed consent will also be obtained from survey participants. The research team will explain the study and its potential risks and benefits to participants who are not able to read on their own. Thumbprints will be collected for informed consent from such participants. During the pretesting phase, we will assess whether monetary compensation is required for the survey participants. The ethical approval of the study has been obtained from the National Bioethics Committee Pakistan (4-87/NBCR-1048/23/1256), Institutional Research and Ethics Forum, Rawalpindi Medical University (725/IREF\RMU\2024), and the Institutional Review Board of the University of Texas (HSC-SPH-23-1089).

## Results

Phases 1 and 2 of the study were completed from January to June 2024. Phase 3 of the study is currently in progress to collect data from primary, secondary, and tertiary health care facilities of Rawalpindi district, Pakistan. We will analyze the survey data by December 2024, followed by report writing and dissemination of results by March 2025.

A thorough quantitative and qualitative analysis will be conducted, which will be aimed at publication in the form of a technical report.

The findings of the study will be disseminated to the scientific community through publications and conference presentations. A dissemination workshop will be organized with the stakeholders of Pakistan to disseminate HSR findings specific to primary, secondary, and tertiary care hospitals of Rawalpindi district, Pakistan.

## Discussion

### Expected Outcomes

This study aims to develop a universal understanding of HSR alongside robust measurement methodology and tools in the context of L&MICs. We believe new domains and/or elements of HSR may be added to the current HSR framework, based on the Delphi process. The revised HSR framework will contribute to global literature and offer a blueprint for other countries to assess and improve HSR and the overall health of the population.

The bulk of prior work on HSR has used the WHO tool without modifications [18,22,25]. Previous efforts to improve the concept of HSR [19,23,26] have not systematically reviewed the current body of evidence or taken a stepwise approach to incorporate views and suggestions of global and L&MICs public health experts, nor have they included pilot-testing of the measurement tool. This study aims to pioneer a robust methodology for assessing HSR from both “demand” and “supply” sides using a mixed methods approach. We will also determine the importance of each HSR domain from the perspectives of global experts (through the Delphi process) and study participants (through a facility-based survey). Comparing the importance of HSR domains from these two diverse perspectives will help unfold a deeper understanding of HSR and identify the needs of people in L&MICs.

The findings from this study will be useful for policy makers to support evidence-informed decision-making. HSR measures the level of user satisfaction with health services and not the system’s response to health needs, which is included in health outcomes [57]. Investing in HSR is a cost-effective strategy for improving health systems utilization and access to services. It also helps early diagnosis and management of diseases and improves treatment compliance. *“*What cannot be measured cannot be improved*,”* as the famous quote by William Thomson says. This study will assess HSR from multiple angles and include the voice of the community and service providers. It will also highlight specific domains of HSR and action points for policy makers to improve HSR. The results of the study will translate into guidelines and recommendations for policy makers to improve HSR and enhance overall population health.

### Strengths and Limitations

Although the greatest strength of this study is its contribution to the global literature, it is not without limitations. Gray literature was not included in the systematic review, and the study was confined to English language. Further bodies of knowledge exist in other languages and outside of traditional publishing channels, which could have led to useful insights for revising the conceptual framework of HSR in the context of L&MICs. The questionnaire will be administered in “Urdu,” which is the national language of Pakistan. There is a possibility that some participants speak other subnational languages and would be left out of the study. Another limitation is not including adolescents (13 to 18 years of age) in the facility-based quantitative survey. They were excluded for feasibility, as assent alongside consent would have been required. Additionally, we have no reason to hypothesize that HSR would differ for adolescents compared to children and adults. Beyond that, we foresee a positive picture of responsiveness being narrated by hospital managers and service providers (ie, supply side). We have tried to limit information bias in the results by introducing an observation checklist that will be completed by the research team. The tool will be tested in only 1 district of the Punjab province. The results would be generalizable to Punjab but would need to be interpreted cautiously for other provinces. Lastly, we intend to formulate a measurement tool applicable to all L&MICs. However, different countries have varied health systems, which are deeply rooted in diverse sociocultural contexts. Countries will always differ from one another, as there is no “average country.” Thus, we recommend other researchers to pilot-test the conceptual framework and measurement tool and tailor them to suit their specific context before using them widely.

## Appendix

Multimedia Appendix 1 Systematic review.

Multimedia Appendix 2 Measurement tool.

Multimedia Appendix 3 Interview guides and observation checklist.

Multimedia Appendix 4 Sample size calculations.

## Abbreviations

HRH: Human Resource for Health
HSR: health systems responsiveness
L&MIC: low- and middle-income country
WHO: World Health Organization

## Data Availability

The modified HSR conceptual framework and measurement tool for L&MICs will be published and freely available online.
