# Supplementary material for: Rethinking Health Systems Responsiveness in Low- and Middle-Income Countries: Validation Study
Source: JMIR Res Protoc. 2024 Sep 18;13:e59836. doi: 10.2196/59836 (PMC11447431; doi:10.2196/59836)
Supplement: Multimedia Appendix 1 [file resprot_v13i1e59836_app1.docx]

**RETHINKING HEALTH SYSTEMS RESPONSIVENESS IN LOW-AND MIDDLE-INCOME COUNTRIES**

***Systematic/ Desk Review***

We carried out a desk review to gather available bodies of literature pertaining to the concept of responsiveness of health systems. We started with the WHO framework of responsiveness based on 8 domains viz; respect for dignity, respect for autonomy, respect for confidentiality, prompt attention, quality of basic amenities, access to social support networks during care, choice of provider and clarity of communication. We also kept the WHO tool used in the World Health Survey of 2002 as the baseline scale. This tool was administered to communities to assess health systems responsiveness. The questionnaire had questions and vignettes pertaining to each domain. We did not include vignettes in our tool. We intend to formulate a tool which is universally applicable to all L&MICs. It was thought that vignettes should be culturally sensitive for better understanding and interpretation of respondents. Local adaptation of minor phrases such as “across the road” is warranted as the width of roads may differ across countries. Although vignettes can potentially provide more robust results, they were omitted from the tool for universal application and maintaining uniformity across countries.^(1)^ It was pre-decided to edit the *framework* and *tool* according to the literature. We will test the framework via Delphi process and the tool will be pilot tested in Pakistan. Although changes in the tool were made in accordance with the desk review, it will be re-visited after the Delphi process by PI and co-PIs of the study *(explained in detail in the following sections)*. All the suggested changes will be re-visited and modified (if necessary) according to experts’ opinion on the conceptual framework. The study team will ensure that the tool is properly aligned with the final conceptual framework.

## Desk Review: Specific Objectives

The specific objectives of desk review were to,

- build on the WHO conceptual framework of health systems responsiveness
- modify and upgrade WHO assessment tool of responsiveness of health systems, in the context of low-and middle-income countries (L&MICs)

## Desk Review: Methods

Published records were searched using systematic search strategy and methods, to gather relevant literature. Three representative articles were identified to guide literature search:

- Murray, Christopher J. L, Evans, David B & World Health Organization. Global Programme on Evidence for Health Policy. (‎2003)‎. Health systems performance assessment : debates, methods and empiricism / edited by Christopher J. L. Murray, David B. Evans. World Health Organization. <https://apps.who.int/iris/handle/10665/42735>
- Njeru MK, Blystad A, Nyamongo IK, Fylkesnes K. A critical assessment of the WHO responsiveness tool: lessons from voluntary HIV testing and counselling services in Kenya. BMC Health Serv Res. 2009 Dec 22;9:243. doi: 10.1186/1472-6963-9-243. PMID: 20028540; PMCID: PMC2811110.
- Mirzoev T, Kane S. What is health systems responsiveness? Review of existing knowledge and proposed conceptual framework. BMJ Glob Health. 2017 Oct 31;2(4):e000486. doi: 10.1136/bmjgh-2017-000486. PMID: 29225953; PMCID: PMC5717934.

The search engines chosen for systematic search were PubMed and Google Scholar. PubMed was incorporated as a source of biomedical literature and complemented by Google Scholar. The search string was built around constructs of (a) health systems and (b) responsiveness. Mesh terms were included for *health systems*. The components (respect for persons & client orientation) and domains of responsiveness were not included separately as search terms, as the databases yielded non-health related records; thus *responsiveness* was included as such to retain its WHO concept. Table 1 details the search terms for each search engine, built by Mesh terms. Records were included if they were published after 1990, were in English language and related to human research. Time filter was applied as responsiveness of health systems as a concept emerged in late 1990s. PubMed and Google Scholar yielded 715 and 129 initial hits respectively (n= 844). The records were exported to Endnote, and duplicates were removed, dropping the total number of records to 824. It was confirmed that all three articles were found with the search string. These records were transferred to JBI Sumari for screening.

| Table 1: Search strategy of systematic review | | | |
| --- | --- | --- | --- |
|  | **Search String** | **Hits** | **Filters** |
| PubMed | ("Health systems" OR "Healthcare System" OR "Healthcare Systems" OR "Health Care System" OR "System, Healthcare" OR "Systems, Healthcare" OR "System, Health Care" OR "Systems, Health Care" OR "Health Care Systems") AND ("Responsiveness") | 715 hits | 1990 and beyond  Humans  English |
| Google Scholar | ("Health systems" OR "Healthcare System" OR "Healthcare Systems" OR "Health Care System" OR "System, Healthcare" OR "Systems, Healthcare" OR "System, Health Care" OR "Systems, Health Care" OR "Health Care Systems") AND ("Responsiveness") | 129 hits | 1990 and beyond  Only Titles (not anywhere in the article) |

Screening was done at three levels: (a) title, (b) abstract and (c) full text screening. The criterion for each level is detailed in table 2.

| **Table 2** | **Criteria for title, abstract and full text screening** |
| --- | --- |
| Title screening | The record should address responsiveness of health systems.  The study should have been carried out in L&MICs |
| Abstract screening | The record adds to the conceptual basis of responsiveness of health systems as defined by WHO.  The record presents a critique of the components of responsiveness of health system, outlined by WHO.  The record offers a critical appraisal of the WHO tool to measure responsiveness of health systems.  The record presents a tool to measure responsiveness |
| Full Text Screening | The record should address responsiveness of health systems.  The study should have been carried out in L&MICs.  The record adds to the conceptual basis of responsiveness of health systems as defined by WHO.  The record presents a critique of the components of responsiveness of health system, outlined by WHO.  The record offers a critical appraisal of the WHO tool to measure responsiveness of health systems.  The record presents a tool to measure responsiveness |

Figure 2 shows the Prisma diagram elaborating selection of documents.

Figure 2: Prisma diagram of selection of records for desk review.

Records filtered for abstract review.

n=181

Total records

N= 844

Total articles after removal of duplicates

N = 824

Records identified through PubMed database searching.

n=715

Records identified through Google Scholar database searching.

n=129

Abstracts filtered for full text review.

n=73

Exclusion of Abstracts (n=108)

High income country: n = 22

Article did not address responsiveness according to the WHO concept/ definition: n = 57

Abstract not available: n = 4

Mechanisms to improve responsiveness: n = 2

Study protocol: n = 2

Duplicate study: n = 21

Exclusion of Full texts (n= 54)

Records addressed responsiveness vignettes: n=3

High income country: n=3

Relevance: n = 14

Determinants of responsiveness: n = 5

Application of tool: n = 11

Repetition: n = 3

Full text not available: n = 8

Full text articles included.

n=19

Exclusion Criteria for Title Screening

1. The record should address responsiveness of health systems.
2. The study should have been carried out in L&MICs

Number of records excluded n=643

Data was extracted from records in the categories of (a) modification to WHO conceptual framework of responsiveness, (b) modification to WHO assessment tool of responsiveness. Appendix I shows the data extraction form.

## Desk Review: Results

A total of 19 articles were found eligible for the review.^(2-7, 1, 8-19)^ Table 3 shows the distribution of articles according to study type. Figure 3 shows the distribution of articles according to study setting.

Table 3: Distribution of articles of desk review according to study type (n=19).

| **Study Type** | **Number of articles** |
| --- | --- |
| Quantitative | 10 |
| Qualitative | 5 |
| Mixed methods | 2 |
| Theme paper | 1 |
| Review article | 1 |
| ***Total*** | ***19*** |

Figure 3: Study setting of selected articles.

### Modification to WHO Concept of Responsiveness

Five records contributed to modifying the WHO conceptual framework of responsiveness based on eight domains viz; respect for dignity, respect for autonomy, respect for confidentiality, prompt attention, quality of basic amenities, access to social support networks during care, choice of provider and clarity of communication.^(5, 1, 11, 10, 14)^ These articles suggested to add further domains to enhance the concept of responsiveness of health systems.^(5, 1, 11, 14, 16)^ We are adding the following domains to the responsiveness framework:

1. Guidance
2. Trust
3. Coordination and continuity of care
4. Financial sensitivity

***Guidance ^(5, 1, 11, 16)^***

The authors of the selected articles suggested that general information on disease prevention and healthy lifestyle – which may or may not be directly related to the patient's disease - is crucial and should be included in responsiveness. Health providers should provide general health promotion and disease prevention-related information to patients, besides explanation and advice particularly related to their health condition. Another article indicates to incorporate this aspect in *autonomy*; however, we suggest keeping it as a part of *guidance.^(1)^* Health providers are also required to provide clear guidance on post treatment follow-up of patients and give them a follow-up plan. The patient should know when to meet the doctor again, the red flags of disease progression, how to reach the doctor in case of an emergency (a phone number to call), follow-up costs, what the patient should come up with (tests/ documents) at the time of follow-up etc.^(5)^ Furthermore, health providers should also explain to the patients how to access medicines, and prescribed diagnostic services including imaging, laboratory testing etc.^(16)^

***Building Trust ^(5-7, 14)^***

Trust, in the context of health system responsiveness is defined as, “The healthcare provider advised for maximizing the patient's benefit, not for maximizing his/ her own benefit." The health care providers should be service oriented and not demonstrate business like behavior e.g., telling the patient to get tested from a specific diagnostic center, encouraging to buy medicines of a specific pharmaceutical company, taking money from patients forcibly, telling the patient under consultation of a govt. doctor to go to a private clinic, etc. It is also important that the healthcare providers are not involved in any unethical/ illegal activities e.g., taking money from patients against free services; bringing patients with the help of brokers; collusion with diagnostic centers, accepting gifts from medical representatives (and prescribing medicine of that company); taking advantage from brokers (utilizing in hospital, utilizing for personal work) and so on. Feedback and accountability mechanisms are vital in building the trust of patients.^(6)^

***Coordination and Continuity of Care*^(10, 11, 14)^**

Continuity of care refers to the extent to which care progresses smoothly as the patient moves across different healthcare providers and sectors. Patients expect healthcare providers to coordinate and discuss with other experts in case of confusion related to the disease. Coordination with other providers is considered essential to maintain continuity of care for the patients. It is also expected that the provider will refer the patient to another doctor immediately, if he cannot diagnose or treat the disease himself or the medical condition is out of his scope of expertise.

***Financial sensitivity*^(5, 7)^**

It is expected that healthcare providers should treat patients according to their financial capacity, as far as possible. The healthcare providers should directly or indirectly (such as asking about profession) ask patients about their income and to assess if they would be able to bear the financial cost of treatment. They should tell the patients how much capital would be needed to complete the treatment; how long the treatment may continue; what impact the patient would be able to put on his ability of income during and after receiving treatment. Prescribing low cost antibiotics; taking less or no consultation fee (in case of private doctors); helping patients from ‘poor fund’; helping for getting free medicines from the hospital (in case of government doctors); giving time and advice to collect money; focusing on the history and physical examination to avoid investigation; prescribing the essential tests only; cutting the commission paid to the doctor for each test; recommending that treatment method to the patient which saves money (meeting the nutritional needs from domestic sources, suggesting the pregnant woman to spend money for nutritious food instead of repeated ultra-sonography etc.) are some strategies under financial sensitivity, as part of responsiveness.

***Modification of Existing Domains (Clarity of Communication & Prompt Attention to Care)***

“Prompt attention” is one of the domains of responsiveness and includes having short travel and waiting times for accessing care. We suggest changing “prompt attention” to “access to care,” and retaining its elements. We suggest adding “attention” to “clarity of communication.” Attention captures if the healthcare provider gave appropriate amount of time to the patient, with enough time to ask questions and clarify ambiguities. There were no avoidable interruptions during the visit e.g., attending unnecessary telephone calls, texting, singing, chatting with peers etc. It also includes insightful listening, to know if the healthcare providers attended and responded with deep understanding to the patients.^(7, 10, 17)^

One of the most important impediments for patients to understand doctor's advice is the medical terminology (jargon), professional language etc. The healthcare providers should avoid such language or explain it if used and use a language that is understandable by the patient.^(5, 11)^ We suggest to add this element to *clarity of communication.*

| **Domains of HSR** | **Definition/ Concept** | **Elements of the domain** | | | | | |
| --- | --- | --- | --- | --- | --- | --- | --- |
| **1. Respect for Dignity** | At the extreme, this means not sterilizing individuals with a genetic disorder or locking up people with communicable diseases, which would violate basic human rights. More generally, it means not humiliating or demeaning patients. | Treatment with respect by health care staff. Patients should be welcomed at the health care unit, addressed respectfully at all times, not shouted at or abused. | Privacy during examination and treatment | Safeguarding of human rights (e.g., liberty to free movement for patients of leprosy, tuberculosis) |  |  |  |
| **2. Respect for Autonomy** | This refers to freedom of patients to participate in choices about their own health. This includes helping choose what treatment to receive or not to receive. | Right of an individual to information on his/her disease and alternative treatment options. | Right to be consulted about treatment. | Informed consent in the context of testing and treatment. | The right of patients of sound mind to refuse treatment. |  |  |
| **3. Respect for Confidentiality** | It refers to the right to determine who has access to one’s personal health related information. | Conducting consultations with the patients in a manner that protects their privacy. | Safeguarding the confidentiality of information provided by the patient, and information relating to an individual’s illness (except where such information needs to be given to another health care provider) |  |  |  |  |
| **4. Quality of Basic Amenities** | It refers to the quality of the environment in which health care is provided. | Clean water Clean Toilets  Clean Linen | Sufficient ventilation (fresh air) | - Clean surroundings  - Regular procedures for cleaning & maintenance of hospital buildings and premises | Healthy & edible food | Adequate furniture and seating |  |
| **5. Access to Social Support Networks during Care** | It refers to provision of social needs for people receiving healthcare. Only applies to people receiving inpatient care. | Patients should be allowed visits by relatives and friends | Provision of food and other consumables by relatives and friends, if not provided by the hospital | Religious practices that do not prove a hinderance to hospital activities or hurt the sensibilities of other  Individuals. | Access to newspapers, radio and TV | **Support to the family of patients (caregivers)^(1)^ | Social financial networks |
| **6. Choice of Provider** | It refers to freedom to select which individual or organization delivers one’s care. | Patients should be able to reach health services of  choice without too much difficulty | Within a health care unit,individuals should be able to  choose their health care provider | Individuals should be able to get a second opinion in  cases of severe or chronic illness or surgery | Individuals should be able to get general and specialist  care as appropriate | **Choice of gender of the provider^(10)^ |  |
| **7. Prompt Access to care*** | Health care facilities should be geographically accessible – taking account of distance, transport, and terrain. People should also be promptly given care once in the healthcare setting. | Patients should be entitled to rapid care in emergencies | Patients should be entitled to care within reasonable time periods even in the case of non-emergency health care problems | Waiting times for consultation and treatment should be reasonable. |  |  |  |
| **8. Attention & Clarity of Communication** ^#^ | Encompasses if proper attention was given to the patient by healthcare providers and if there was appropriate communication | Clarity in conveying information and evoking understanding | Providing time for patients to understand their symptoms and to ask questions. **Enough time.^(10)^** | Insightful listening **^(10)^ | No interruptions during consultation (Unnecessary calls, texting, chatting, singing etc)**^(7)^ | Not using jargon**^(5)^ | Asking patient if he/ she understood the explanation (quality of counselling)^(11)^ |
| **9. Building Trust^(5-7, 14)^** ^##^ | The healthcare provider advised for maximizing the patients benefit, not for maximizing his/ her own benefit. | Service oriented, not business-like behavior  - Being asked to do tests from specified diagnostic centers  -Visit them privately (by public sector physician)-Moonlighting | Not being involved in illegal activities  -Bringing patients in own private clinics  -Accepting gift from pharmaceutical representatives --prescribing substandard medicine | Mechanisms of accountability | Earning trust of patients |  |  |
| **10. Guidance** ^(5, 1)# #^ | General information on maintaining healthy lifestyle and specific guidance on the disease under consideration | Information & suggestions on healthy lifestyle in general (e.g., smoking cessation, physical activity, healthy diet etc.) | Information and suggestions on disease prevention | Facilitating follow-up | Explaining access to medicines and diagnostic services **^(16)^** |  |  |
| **11. Financial Sensitivity^(5, 7)^** ^##^ | Refers to how mindful healthcare providers are to the financial burden put on patients due to illness | Trying to understand socio-economic status of the patient. | Considering socio-economic status of the patient in discussing management options | Informing the cost of treatment/financial counselling. | Providing financial assistance if needed, referring to organizations or individuals that can provide financial assistance |  |  |
| **12. Coordination and Continuity Of Care^(5, 10, 11, 14)^**^##^ | It refers to the extent to which care progresses smoothly as the patient moves across different healthcare providers and sectors | Continuity of care  -Communication between providers | Referral services |  |  |  |  |

*Previously, this domain was “prompt attention” in the WHO framework of responsiveness

** New added items in the original WHO domains of responsiveness

^#^ *Attention* has been added to c*larity of communication*

^##^ Newly added domains^.^ The green boxes are new additions.

We have modified WHO HSR tool with the help of the desk review. However, these revisions are not final. The current modifications are detailed below.

### Modifications to the WHO Responsiveness Assessment Tool

Sixteen records from the desk review contributed to modifying the WHO responsiveness assessment tool. ^(2-7, 1, 8-13, 17-19)^ This is a community-based questionnaire, administered to adults, which asks about their past experiences of engagement with out-patient and in-patient components of health system. We have modified the questionnaire according to the modified conceptual framework. This questionnaire will be re-visited by the study team, after the completion of Delphi technique on the conceptual framework.

**Addition of Domains to the Questionnaire**

We have added four domains to the WHO responsiveness tool, based on the modified conceptual framework (Table 4). These are building trust, guidance, financial sensitivity, and coordination & continuity of care. Each domain has specific questions followed by a composite question (scale of 1 to 5) to elaborately understand it (Appendix II: Modified Measurement tool). The importance of these domains is also assessed by adding one question for each in the last module of the *questionnaire* (Appendix II)*.*

**Modification of Items in the Questionnaire**

It was suggested to add “support to the family of patients” in *access to social support networks during care*.^(1)^ We added “choice of gender” in the domain of *choice of provider,* as this is an important consideration for many diseases specially affecting women, e.g. breast cancer. The domain of “prompt attention” was modified to “prompt access to care,” as it does not capture aspects of “attention,” which was added in clarity of communication (*attention and clarity of communication)*.

Furthermore, WHS uses a Likert scale (Very good, Good, Moderate, Bad, Very bad) to capture responses of questions for each domain. We changed it to a scale of 1 to 5, with 5 being the optimum response. This was changed after consultation with the research team, as it was thought that the people of L&MICs are rarely able to differentiate between adjacent items of a Likert scale. A scale of 0 to 10 has most frequently been used in the literature,^(3, 4, 9, 11)^ however, it was felt that people tend to choose categories around the middle on such a scale and variability is rarely captured. Categories of “don’t know” and “no response” were also added to each question.

### Limitations of the Desk Review

Our search did not include grey literature and was confined to English language. Further bodies of knowledge exist in other languages which could have added useful insights.^(20)^

**References**

1. Murray, Christopher J. L, Evans, David B & World Health Organization. Global Programme on Evidence for Health Policy. (‎2003)‎. Health systems performance assessment : debates, methods and empiricism / edited by Christopher J. L. Murray, David B. Evans. World Health Organization. <https://apps.who.int/iris/handle/10665/42735>.

2. Adelabu A, Akinyemi O, Adebayo A, Oladokun B. Assessment of the level and distribution of health system responsiveness in Oyo State, Nigeria. BMC Health Serv Res. 2022 Jul 12;22(1):905.

3. Peltzer K, Phaswana-Mafuya N. Patient experiences and health system responsiveness among older adults in South Africa. Glob Health Action. 2012 Nov 27;5:1-11. doi: 10.3402/gha.v5i0.18545. PMID: 23195515; PMCID: PMC3509423.

4. Rahman MHU, Singh A, Madhavan H. Disability-based disparity in outpatient health system responsiveness among the older adults in low- to upper-middle-income countries. Health Policy Plan. 2019 Mar 1;34(2):141-150. doi: 10.1093/heapol/czz013. PMID: 30891591.

5. Joarder T, George A, Sarker M, Ahmed S, Peters DH. Who are more responsive? Mixed-methods comparison of public and private sector physicians in rural Bangladesh. Health Policy Plan. 2017 Nov 1;32(suppl_3):iii14-iii24. doi: 10.1093/heapol/czx111. PMID: 29149312.

6. Topp SM, Chipukuma JM. A qualitative study of the role of workplace and interpersonal trust in shaping service quality and responsiveness in Zambian primary health centres. Health Policy Plan. 2016 Mar;31(2):192-204. doi: 10.1093/heapol/czv041. Epub 2015 May 20. PMID: 25999586; PMCID: PMC4748128.

7. Joarder T, George A, Ahmed SM, Rashid SF, Sarker M. What constitutes responsiveness of physicians: A qualitative study in rural Bangladesh. PLoS One. 2017 Dec 18;12(12):e0189962. doi: 10.1371/journal.pone.0189962. PMID: 29253891; PMCID: PMC5734771.

8. Amani PJ, Tungu M, Hurtig AK, Kiwara AD, Frumence G, San Sebastián M. Responsiveness of health care services towards the elderly in Tanzania: does health insurance make a difference? A cross-sectional study. Int J Equity Health. 2020 Oct 12;19(1):179. doi: 10.1186/s12939-020-01270-9. PMID: 33046058; PMCID: PMC7549195.

9. Valentine N, Darby C, Bonsel GJ. Which aspects of non-clinical quality of care are most important? Results from WHO's general population surveys of "health systems responsiveness" in 41 countries. Soc Sci Med. 2008 May;66(9):1939-50. doi: 10.1016/j.socscimed.2007.12.002. Epub 2008 Mar 3. PMID: 18313822.

10. Forouzan AS, Ghazinour M, Dejman M, Rafeiey H, San Sebastian M. Testing the WHO responsiveness concept in the Iranian mental healthcare system: a qualitative study of service users. BMC Health Serv Res. 2011 Nov 25;11:325. doi: 10.1186/1472-6963-11-325. PMID: 22115499; PMCID: PMC3280196.

11. Njeru MK, Blystad A, Nyamongo IK, Fylkesnes K. A critical assessment of the WHO responsiveness tool: lessons from voluntary HIV testing and counselling services in Kenya. BMC Health Serv Res. 2009 Dec 22;9:243. doi: 10.1186/1472-6963-9-243. PMID: 20028540; PMCID: PMC2811110.

12. Asefa G, Atnafu A, Dellie E, Gebremedhin T, Aschalew AY, Tsehay CT. Health System Responsiveness for HIV/AIDS Treatment and Care Services in Shewarobit, North Shewa Zone, Ethiopia. Patient Prefer Adherence. 2021 Mar 9;15:581-588. doi: 10.2147/PPA.S300825. PMID: 33727803; PMCID: PMC7955722.

13. Liabsuetrakul T, Petmanee P, Sanguanchua S, Oumudee N. Health system responsiveness for delivery care in Southern Thailand. Int J Qual Health Care. 2012 Apr;24(2):169-75. doi: 10.1093/intqhc/mzr085. Epub 2012 Jan 2. PMID: 22215759.

14. Mirzoev T, Kane S. What is health systems responsiveness? Review of existing knowledge and proposed conceptual framework. BMJ Glob Health. 2017 Oct 31;2(4):e000486. doi: 10.1136/bmjgh-2017-000486. PMID: 29225953; PMCID: PMC5717934.

15. Negash WD, Tsehay CT, Yazachew L, Asmamaw DB, Desta DZ, Atnafu A. Health system responsiveness and associated factors among outpatients in primary health care facilities in Ethiopia. BMC Health Serv Res. 2022 Feb 24;22(1):249. doi: 10.1186/s12913-022-07651-w. PMID: 35209882; PMCID: PMC8867670.

16. Amani PJ, Hurtig AK, Frumence G, Kiwara AD, Goicolea I, San Sebastiån M. Health insurance and health system (un) responsiveness: a qualitative study with elderly in rural Tanzania. BMC Health Serv Res. 2021 Oct 22;21(1):1140. doi: 10.1186/s12913-021-07144-2. PMID: 34686182; PMCID: PMC8532322.

17. Forouzan AS, Rafiey H, Padyab M, Ghazinour M, Dejman M, Sebastian MS. Reliability and validity of a Mental Health System Responsiveness Questionnaire in Iran. Glob Health Action. 2014 Jul 28;7:24748. doi: 10.3402/gha.v7.24748. PMID: 25079288; PMCID: PMC4116621.

18. Kapologwe NA, Kibusi SM, Borghi J, Gwajima DO, Kalolo A. Assessing health system responsiveness in primary health care facilities in Tanzania. BMC Health Serv Res. 2020 Feb 10;20(1):104. doi: 10.1186/s12913-020-4961-9. PMID: 32041609; PMCID: PMC7011252.

19. Ugurluoglu O, Celik Y. How responsive Turkish health care system is to its citizens: the views of hospital managers. J Med Syst. 2006 Dec;30(6):421-8. doi: 10.1007/s10916-005-9006-8. PMID: 17233154.

20. Shulgina SV. The health system responsiveness: definition, structure, concepts, methodological approaches to research. [Russian]. Ekologiya Cheloveka/Hum Ecol 2015;2:27–32.
