# Supplementary material for: Rethinking Health Systems Responsiveness in Low- and Middle-Income Countries: Validation Study
Source: JMIR Res Protoc. 2024 Sep 18;13:e59836. doi: 10.2196/59836 (PMC11447431; doi:10.2196/59836)
Supplement: Multimedia Appendix 2 [file resprot_v13i1e59836_app2.docx]

|  | **Patient Questionnaire for Healthcare Facility Based Assessment of Responsiveness** |  |  |
| --- | --- | --- | --- |
|  | ***NOTE: The newly added questions are in yellow boxes.** |  |  |
|  | (This is an interviewer administered questionnaire for patients. The patients will be recruited from healthcare facilities. The patient should be 18 years or above and should have sought inpatient or outpatient care from the healthcare facility in the past 7 days. These are exit interviews of the patients. Patients will be recruited from the point of exit from outpatient department or when they are discharged in case of receiving inpatient care. The participants could also be the parents of children who have sought inpatient or outpatient care from the healthcare facility in the past 7 days. Read all options to the respondent except for Refuse and Don’t Know (DK). If a question does not apply to the respondent, circle the option Not Applicable (NA).) | |  |
|  | *These questions are about your [or your child's] experiences of getting health care from this healthcare facility during your current visit (for outpatient) or healthcare facility stay (for in patient).* | |  |
|  | Department from which the participant was recruited | Medicine  Surgery  Gynae/ Obs  Pediatrics  Others |  |
|  |  |  |  |
|  | **Questions** | **Response** | **Skip Pattern** |
|  | **MODULE 1: BASICS** |  |  |
| **Q1** | Have you or your child (under 12 years of age) received any health care from _______ healthcare facility in the last 7 days? | Yes  No | *If 0, the participant in ineligible for the study* |
| **Q2** | Is this health facility your usual place of seeking health care? | Yes  No |  |
| **Q3** | Did you [or your child] get healthcare from this health facility at an outpatient department? | Yes  No | If 0, skip to Q5 |
| **Q4** | Which reason best describes why you [or your child] needed healthcare? | High fever, severe diarrhoe or cough  Immunization  Antenatal consultattion Family planning Childbirth Dental care Arthritis Asthma Heart disease Bodily injury Minor surgery Other | skip to Q9 |
| **Q5** | In which inpatient ward did you [or your child] stay e.g. medicine, surgery, gynae/ obs etc.? (Only for secondary and tertiary care healthcare facilities) |  |  |
| **Q6** | Did you [or your child] stay in this healthcare facility for at least one night in the past 7 days? | Yes  No |  |
| **Q7** | Which of the following best describes the reason for your [or your child's] last overnight stay? | High fever, severe diarrhoe or cough Childbirth Arthritis Asthma Heart disease Bodily injury Minor surgery Other |  |
| **Q8** | How long was your [or your child's] stay on this occasion? | ____days |  |
| **Q9** | Which healthcare provider provided you [or your child] care? Select all that apply. | Medical doctor/ specialist Nurse Midwife Dentist  Physiotherapist or chiropractor Other (Please specify) |  |
|  | **RESPONSIVENESS** |  |  |
|  | *The following questions are related to your [or your child's] current experience of visit to the healthcare outpatient/ inpatient department.* |  |  |
| **Q10** | In your opinion, was the [health care provider’s] skill adequate for your [or your child's] treatment? | Yes  No |  |
| **Q11** | In your opinion, was [the health care provider’s] equipment adequate for your [or your child's] treatment? | Yes  No |  |
| **Q12** | In your opinion, were [the health care provider’s] drug supplies adequate for your [or your child's] treatment? | Yes  No |  |
| **Q13** | For your [or your child's] current visit/ healthcare facility stay, how much did you or your household pay for the following (local currency)? [Interviewer: write 0 if the service was free] | ___ |  |
| **Q14** | Health care provider's fees | ….............(local currency) Free  Don’t know  Refused to answer |  |
| **Q15** | Medicines | ….............(local currency) Free  Don’t know  Refused to answer |  |
| **Q16** | Tests | ….............(local currency) Free  Don’t know  Refused to answer |  |
| **Q17** | Transport | ….............(local currency) Free  Don’t know  Refused to answer |  |
| **Q18** | Other | ….............(local currency) Free  Don’t know  Refused to answer |  |
| **Q19** | Did you or your household pay less than the normal health care fees because of a government discount or exemption? | Yes No |  |
|  | **MODULE 2: DOMAINS OF RESPONSIVENESS** |  |  |
|  | **Prompt Access to Care.** The next questions are about how promptly you [or your child] got care. |  |  |
| **Q20** | How did you [or your child] get to the healthcare facility [Interviewer: mark the one used for most of the travel distance] | Private car or motorcycle  Public transport Ambulance Bicycle Walked Other Don’t know  No response |  |
| **Q21** | How long did it take you [or your child] to get from home to the healthcare facility? | ….....Min ….....Hours …......Days …......Weeks Don’t know … No response …. |  |
| **Q22** | For your current healthcare visit/ healthcare facility stay, did you [or your child] need any laboratory tests or examinations? Some examples of tests or special examinations are blood tests, scans or X rays. | Yes  No  Don’t know … No response …. | If 0, go to Q24 |
| **Q23** | How long did you have to wait before you [or your child] could get the laboratory tests or examinations done? | ….....Min ….....Hours …......Days …......Weeks Don’t know … No response …. |  |
| **Q24** | How would you rate the travelling time to the healthcare provider? | Very good Good Moderate Bad Very bad Don’t know No response |  |
| **Q25** | How would you rate the amount of time you [and your child] waited before being attended to? | Very good Good Moderate Bad Very bad Don’t know No response | For participants from outpatient dept, skip to Q28 |
| **Q26** | For your healthcare facility stay, how long from the time you [or your child] needed healthcare facility care did you wait to be admitted to healthcare facility? | Same day  Less than a week Less than one month Less than 3 months 3 months & more Don’t know … No response …. |  |
| **Q27** | Now, overall on a scale of 1 to 5, how would you rate your [or your child's] experience of getting prompt access to care at the healthcare facility for your current visit? It means: -having short travel times and convenient access to health care facilities - having short waiting times for consultations and healthcare facility admissions with 1 being the worst experience and 5 being the best.... | _______ Don't know …. No response … |  |
|  | **Respect for Dignity: The next questions are about the dignity with which you [or your child] were treated when you sought health care/ healthcare facility stay.** |  |  |
| **Q28** | How would you rate your [or your child's] experience of being greeted and talked to respectfully by the doctors, nurses or health care providers? | Very good Good Moderate Bad Very bad Don’t know No response |  |
| **Q29** | How would you rate your [or your child's] experience of being greeted and talked to respectfully by staff such as receptionists or clerks? | Very good Good Moderate Bad Very bad Don’t know No response |  |
| **Q30** | How would you rate the way your [or your child's] privacy was respected during physical examinations and treatment? | Very good Good Moderate Bad Very bad Don’t know No response |  |
| **Q31** | Do you feel you [or your child] were treated without any discrimination based on gender, race/ ethnicity, socio-economic status etc.? | Yes  No  Don’t know … No response …. |  |
| **Q32** | Overall, how would you rate your [or your child's] experience of getting treated with dignity in the healthcare facility for your current visit, on a scale of 1 to 5, with 1 being the worst experience and 5 being the best? Dignity means being shown respect when greeted by and when talking to health care providers and staff, having physical examinations conducted in a way that respects your cultural norms. Would you say it is ... | _______ Don't know …. No response … |  |
|  |  |  |  |
|  | **Respect for Autonomy: As part of your care, decisions are made about which treatments or tests to give. The next questions are about your [or your child's] involvement in decisions about the care and treatment you received in the current visit or healthcare facility stay.** |  |  |
| **Q33** | How would you rate your [or your child's] experience of being involved in making decisions about your health care or treatment? | Very good Good Moderate Bad Very bad Don’t know No response |  |
| **Q34** | How would you rate your [or your child's] experience of doctors, nurses or other healthcare providers seeking your permission before starting any treatment or ordering any tests? | Very good Good Moderate Bad Very bad Don’t know No response |  |
| **Q35** | Were you given information regarding possible risks and benefits, about a medical procedure or treatment, before starting it? Did you understand all the information? | Yes  No  Don’t know … No response …. |  |
| **Q36** | How would you rate your [or your child's] experience of getting information about other types of treatments or tests available? | Very good Good Moderate Bad Very bad Don’t know No response |  |
| **Q37** | Overall, how would you rate your [or your child's] experience of getting involved in making decisions about your care or treatment as much as you wanted in the currnet visit, on a scale of 1 to 5, with 1 being the worst experience and 5 being the best. Being involved in decision making means -being invloved as much as you want in deciding about your health care - freedom to discuss other treatment options or care regimens if you want. Would you it is… | _______ Don't know …. No response … |  |
|  |  |  |  |
|  | **Respect for Confidentiality: The next set of questions are about your [or your child's] experience of confidentiality of information in the healthcare facility, for your current visit.** |  |  |
| **Q38** | How would you rate the way the health services ensured that you [or your child] could talk privately to the health care providers (so other people who you did not want to hear could not overhear what was said)? | Very good Good Moderate Bad Very bad Don’t know No response |  |
| **Q39** | How would you rate the way your [or your child's] personal information was kept confidential? (This means that anyone whom you did not want informed could not find out about your medical conditions.) | Very good Good Moderate Bad Very bad Don’t know No response |  |
| **Q40** | Overall, how would you rate your [or your child's] experience of the way the health services kept information about you confidential, on a scale of 1 to 5, with 1 being the worst experience and 5 being the best. Confidentiality means -having information about your health and other personal information kept confidential and having conversations with health care providers without other people overhearing. Would you say it was.. | _______ Don't know …. No response … |  |
|  |  |  |  |
|  | **Quality of Basic Amenities:** The next questions are about the environment or the surroundings you [or your child] encountered in your current visit. |  |  |
| **Q41** | How would you rate the cleanliness of the rooms inside the healthcare facility? | Very good Good Moderate Bad Very bad Don’t know No response |  |
| **Q42** | How would you rate the cleanliness of the toilets inside the healthcare facility? | Very good Good Moderate Bad Very bad Don’t know No response |  |
| **Q43** | Was there access to soap at all hand washing areas? | Yes  No  Don’t know … No response …. |  |
| **Q44** | How would you rate the amount of space in the waiting and examination rooms? | Very good Good Moderate Bad Very bad Don’t know No response |  |
| **Q45** | Thinking about your current healthcare facility stay, how many people slept in the same room as you? | ___________ |  |
| **Q46** | For your [or your child's] healthcare facility stay how would you rate the amount of space you had? | Very good Good Moderate Bad Very bad Don’t know No response |  |
| **Q47** | Were you [or your child] provided access to clean drinking water at the healthcare facility? | Yes  No  Don’t know … No response …. |  |
| **Q48** | How would you rate the ventilation of the healthcare facility? | Very good Good Moderate Bad Very bad Don’t know No response |  |
| **Q49** | Was there foul smell in the healthcare facility? | Yes  No  Don’t know … No response …. |  |
| **Q50** | How would you rate the facilities provided for people with disabilities in the healthcare facility? | Very good Good Moderate Bad Very bad Don’t know No response |  |
| **Q51** | Now, overall, how would you rate the overall quality of the surroundings, for example space, seating, fresh air and cleanliness of the healthcare facility, on a scale of 1 to 5, with 1 being the worst experience and 5 being the best? | _______ Don't know …. No response … |  |
|  |  |  |  |
|  | **Access to Social Support Networks During Care:** These questions will only be asked for from inpatient patients. |  | For participants from the outpatient dept, skip to Q58 |
| **Q52** | For your [or your child's] healthcare facility stay, how would you rate the ease of having family and friends visit you? | Very good Good Moderate Bad Very bad Don’t know No response |  |
| **Q53** | For your [or your child's] healthcare facility stay, how would you rate your experience of staying in contact with the outside world when you were in healthcare facility (e.g., access to newspaper, TV, internet)? | Very good Good Moderate Bad Very bad Don’t know No response |  |
| **Q54** | How would you rate the ease of having outside food and other consumables by relatives and friends if not provided by the healthcare facility? | Very good Good Moderate Bad Very bad Don’t know No response |  |
| **Q55** | How would you rate the support offered to family and relatives, during the healthcare facility stay? | Very good Good Moderate Bad Very bad Don’t know No response |  |
| **Q56** | How would you rate the ease of continuing religious practices in the healthcare facility that do not prove to be a hinderance to the healthcare facility activities? | Very good Good Moderate Bad Very bad Don’t know No response |  |
| **Q57** | Overall, on a scale of 1 to 5, with 1 being worst and 5 best experience, how would you rate the overall access to social support networks provided by the healthcare facility? | _______ Don't know …. No response … |  |
|  |  |  |  |
|  | **Choice of Provider:** The next questions are about the choice of health care providers you had for your [or your child's] current visit. |  |  |
| **Q58** | How would you rate the freedom you had to choose your [or your child's] health care provider? | Very good Good Moderate Bad Very bad Don’t know No response |  |
| **Q59** | Were you given a choice to choose the gender of your [or your child's] healthcare provider? | Yes  No  Don’t know … No response …. |  |
| **Q60** | Overall, on a scale of 1 to 5, with 1 being the worst experience and 5 being the best, how would you rate your experience of being able to use a health care provider or service of your choice for yourself or your child. Choice means -being able to choose your health care provider (place or person) -being able to consult for a second opinion or with a specialist if so desired | _______ Don't know …. No response … |  |
|  |  |  |  |
|  | **Attention & Clarity of Communication:** The next questions are about attention provided to you during consultation and how health care providers communicated with you. |  |  |
| **Q61** | Do you think you [or your child] were provided enough time for consultation? | Yes  No  Don’t know … No response …. |  |
| **Q62** | How would you rate your experience of getting enough time to ask questions about your [or your child's] health problem or treatment? | Very good Good Moderate Bad Very bad Don’t know No response |  |
| **Q63** | How would you rate the experience of how doctors, nurses or other health care providers listened carefully to you? | Very good Good Moderate Bad Very bad Don’t know No response |  |
| **Q64** | Do you feel that the healthcare provider understood all your concerns? | Yes  No  Don’t know … No response …. |  |
| **Q65** | Were there any interruptions during consultation such as attending unnecessary calls, texting, singing, chatting with peers etc.? | Yes  No  Don’t know … No response …. |  |
| **Q66** | How would you rate the experience of how clearly health care providers explained things to you in a way/ language you could understand? | Very good Good Moderate Bad Very bad Don’t know No response |  |
| **Q67** | How would you rate the quality of counselling provided to you [or your child] by the healthcare providers? | Very good Good Moderate Bad Very bad Don’t know No response |  |
| **Q68** | How would you rate your experience of how well health care providers communicated with you and gave you attention in the current visit. Communication means having the health care providers explain things in a way you can understand and having enough time to ask questions if you don't understand something. Rate on a scale  on a scale of 1 to 5, with 1 being the worst experience and 5 being the best.. | _______ Don't know …. No response … |  |
|  |  |  |  |
|  | **BUILDING TRUST (The next few questions are about the behavior of healthcare providers, to build your trust)** |  |  |
| **Q69** | Did the healthcare providers suggest a specific diagnostic facility for your [or your child's] tests, which you felt was for personal motives? | Yes  No  Don’t know … No response …. |  |
| **Q70** | Did the healthcare providers suggest and encourage to buy medicines from a specific pharmaceutical company, which you felt was for personal motives? | Yes  No  Don’t know … No response …. |  |
| **Q71** | Were you told by the healthcare providers to go to a specific private clinic/ healthcare facility for better treatment and management, which you felt was for personal motives? | Yes  No  Don’t know … No response …. |  |
| **Q72** | Do you trust that your consultation/ treatment was not influenced by any motives other than your medical well-being. | Yes  No  Don’t know … No response …. |  |
| **Q73** | Are there any feedback mechanisms to hold providers and healthcare facility accountable ? | Yes  No  Don’t know … No response …. |  |
| **Q74** | Overall on a scale of 1 to 5 with 1 being the worst experience and 5 being the best, how much do you think that your consultation/ treatment was not effected by any personal motives other than your [or your child's] wellbeing? | _______ Don't know …. No response … |  |
|  |  |  |  |
|  | **GUIDANCE** |  |  |
| **Q75** | On a scale of 1 to 5, how do you rate the suggestions and guidance on disease prevention and health promotion given to you in general (for example, using sanitary latrines, habit of hand washing, vaccinating children, giving breast milk to the infants, physical activity or exercise, giving up smoking, general cleanliness, avoiding fatty foods, using germ-free water, eating nutritious food etc.), with 0 being no guidance. | _______ Don't know …. No response … |  |
| **Q76** | On a scale of 1 to 5, how would you rate the follow-up plan provided by the healthcare provider for the next visit? The follow-up plan includes instructions about when to visit, follow up costs, informing the patients about tests and documentation that they need to bring.etc. 1 being no plan provided and 5 being a detailed plan. | _______ Don't know …. No response … |  |
| **Q77** | On a scale of 1 to 5, how would you rate the explanation and elaboration of cause, diagnosis, prognosis, treatment (including side effects of drugs), management, and preventive aspects of disease provided to you by the healthcare provider? 1 being no explanation and 5 being complete information. (counselling) | _______ Don't know …. No response … |  |
| **Q78** | On a scale of 1 to 5, how would you rate the guidance provided to you regarding accessing medicines and prescribed diagnostic services including imaging, laboratory testing etc.? 1 being no guidance and 5 being complete information. | _______ Don't know …. No response … |  |
| **Q79** | Now, overall, on a scale of 1 to 5, how would you rate guidance provided to you by the service providers with 1 being no guidance and 5 being complete guidance? | _______ Don't know …. No response … |  |
|  |  |  |  |
|  | **FINANCIAL SENSITIVITY** |  |  |
| **Q80** | Did the healthcare provider tell you the cost to complete the treatment, how long treatment may continue and what would be the impact on the earning ability of the patient after completion of the treatment? | Yes  No  Don’t know … No response …. |  |
| **Q81** | Did the healthcare provider ask you about your profession and/ or income, or if you would be able to bear the cost of treatment? | Yes  No  Don’t know … No response …. |  |
| **Q82** | On a scale of 1 to 5, how would you rate the financial assistance provided to you by the healthcare provider and healthcare facility. This includes prescribing low cost antibiotics, taking less or no consultation fee (in case of private doctors), providing financial assistance to the poor patients, helping in getting free medicines from the healthcare facility (in case of government doctors), giving time and advice to obtain money for treatment, trying to focus on the history and physical examination to avoid investigation, prescribing the essential tests only, deducting the commission paid to the doctor for each test, recommending the treatment method that saves money (to meet the nutritional needs from domestic sources, suggesting the pregnant woman to spend money for nutritious food instead of repeated ultra sonography etc.) and so on. | _______ Don't know …. No response … |  |
| **Q83** | Now, overall on a scale of 1 to 5, how would you rate the financial sensitivity of the healthcare provider and healthcare facility with 1 being completely insensitive and 5 being very sensitive? | _______ Don't know …. No response … |  |
|  |  |  |  |
|  | **COORDINATION AND CONTINUITY OF CARE** |  |  |
| **Q84** | Do you think/ did you experience that there was good coordination of course of treatment among different healthcare providers? | Yes  No  Don’t know … No response …. |  |
| **Q85** | In case of inability of the doctor to treat your disease (such as inability to understand X-ray, ECG, ultra sonogram, inability to diagnose skin diseases confidently etc.), the doctor provided treatment after discussing with a related person promptly. | _______ Don't know …. No response … |  |
| **Q86** | In case of inability of the doctor to treat your disease, he explained his limitations, told where to go, wrote the address of the doctor/healthcare facility where to go, and satisfactorily did all the tasks related with referring the patient. | Yes  No  Don’t know … No response …. |  |
| **Q87** | Now, overall on a scale of 1 to 5, how would you rate the coordination, continuity of care and referral services provided by the healthcare providers and the healthcare facility with 1 being poor performance coordination and 5 being perfect performance? | _______ Don't know …. No response … |  |
|  |  |  |  |
|  | **ACCESS TO MEDICINES AND DIAGNOSTIC FACILITIES** |  |  |
|  | Were you provided access to medicines required for the management of your disease? | Yes  No  Don’t know … No response …. |  |
|  | Were you provided access to diagnostic facilities required for the management of your disease? | Yes  No  Don’t know … No response …. |  |
|  | Now, overall on a scale of 1 to 5, how would you rate the access to medicines and diagnostic facilities, provided by healthcare providers and healthcare facility with 1 being no access and 5 being perfect access? | _______ Don't know …. No response … |  |
|  |  |  |  |
|  |  |  |  |
|  | **MODULE 3: DISPARITY IN RESPONSIVENESS** |  |  |
|  | In your visit ( for outpatient) or stay (in-patient) did you feel that you were treated worse by health care providers for any of the following reasons. Because of your: |  |  |
| **Q88** | Sex | _______ Don't know …. No response … |  |
| **Q89** | Age | _______ Don't know …. No response … |  |
| **Q90** | Lack of money | _______ Don't know …. No response … |  |
| **Q91** | Social class | _______ Don't know …. No response … |  |
| **Q92** | Ethnic group or color | _______ Don't know …. No response … |  |
| **Q93** | Type of illness | _______ Don't know …. No response … |  |
| **Q94** | Nationality | _______ Don't know …. No response … |  |
| **Q95** | Religion | _______ Don't know …. No response … |  |
| **Q96** | Disability | _______ Don't know …. No response … |  |
|  | **MODULE 4: IMPORTANCE OF ELEMENTS OF RESPONSIVENESS** |  |  |
| **Q97** | How important is "respectful treatment" to you, on a scale of 1 to 5, with 1 being not important and 5 being extremely important. This means  -being shown respect when greeted by and when talking to health care providers and staff -having physical examinations conducted in a way that respects your cultural norms. Would you say it is ......**[respect for dignity]** | _______ Don't know …. No response … |  |
| **Q98** | How important is "confidentiality of personal information" to you on a scale of 1 to 5, with 1 being not important and 5 being extremely important. This means -having information about your health and other personal information kept confidential -having conversations with health care providers without other people overhearing. Would you say it is.....[**Respect for confidentiality]** | _______ Don't know …. No response … |  |
| **Q99** | How important is "convenient travel and short waiting times" on a scale of 1 to 5, with 1 being not important and 5 being extremely important? This means -having short travel times and convenient access to health care facilities - having short waiting times for consultations and healthcare facility admissions.  - no interruptions during consultation...Would you say it is…[prompt attention] | _______ Don't know …. No response … |  |
| **Q100** | How important is "choice of health care providers" to you on a scale of 1 to 5, with 1 being not important and 5 being extremely important. This means, -being able to choose your health care provider (place or person) -being able to consult for a second opinion or with a specialist if so desired..Would you say it is… [**choice of provider]** | _______ Don't know …. No response … |  |
| **Q101** | How important is "involvement in decision making" to you on a scale of 1 to 5, with 1 being not important and 5 being extremely important. This means -being invloved as much as you want in deciding about your health care - freedom to discuss other treatment options or care regimens if you want. Would you it is….[**respect for autonomy]** | _______ Don't know …. No response … |  |
| **Q102** | How important are "good quality surroundings" to you on a scale of 1 to 5, with 1 being not important and 5 being extremely important? This means  -having enough space, seating and fresh air in the waiting rooms, examination rooms and healthcare facility wards -having a clean facility (including clean toilets). Would you say it is...[Quality of Basic Amenities] | _______ Don't know …. No response … |  |
| **Q103** | How important is "contact with the outside world " to you on a scale of 1 to 5, with 1 being not important and 5 being extremely important? This means -having family and friends visit you as much as you want when you are a patient in healthcare facility -being able to keep in contact with family and friends and to have information about what is happening outside the healthcare facility. Would yo say it is..[Access to Social Support Networks during Care] | _______ Don't know …. No response … |  |
| **Q104** | How important is "attention & clarity of communication" to you on a scale of 1 to 5, with 1 being not important and 5 being extremely important. This means  having the health care providers explain things in a way you can understand -having enough time to ask questions if you don't understand something Would you say it is….**[Attention and Clarity of Communication]** | _______ Don't know …. No response … |  |
| **Q105** | How important is "building trust" to you on a scale of 1 to 5, with 1 being not important and 5 being extremely important. This means that the healthcare providers show service oriented behavior and refrain from un-ethical practices such as advising a specific diagnostic facility or suggesting to buy medicines from a specific pharmaceutical company for personal gains. Existance of feedback and accountability mechanisms is also vital. It is also important that healthcare providers do not use jargon in communicating with patients...**[Building Trust]** | _______ Don't know …. No response … |  |
| **Q106** | How important is "guidance" to you on a scale of 1 to 5, with 1 being not important and 5 being extremely important? This means providing general health promotion and disease prevention advice to patients, explaining disease aspects, and providing with a follow-up plan? **[Guidance]** | _______ Don't know …. No response … |  |
| **Q107** | How important is financial sensitivity to you on a scale of 1 to 5, with 1 being not important and 5 being extremely important? This means that the healthcare provider considers socio-economic status of the patient, suggests management and treatment options accordingly, and makes every possible legitimate effort in helping the patient to bear the cost of treatment...**[Financial Sensitivity]** | _______ Don't know …. No response … |  |
| **Q108** | How important is "coordination and continuity of care" to you on a scale of 1 to 5, with 1 being not important and 5 being extremely important? This means smooth transition from one healthcare provider to another if required and coordination among providers to discuss your disease management? **[Coordination and Continuity of Care]** | _______ Don't know …. No response … |  |
