# Supplementary material for: Rethinking Health Systems Responsiveness in Low- and Middle-Income Countries: Validation Study
Source: JMIR Res Protoc. 2024 Sep 18;13:e59836. doi: 10.2196/59836 (PMC11447431; doi:10.2196/59836)
Supplement: Multimedia Appendix 3 [file resprot_v13i1e59836_app3.docx]

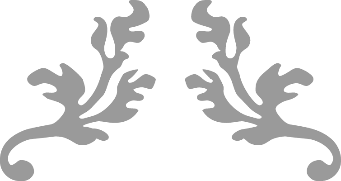


Rethinking Health Systems Responsiveness in Low-and Middle-Income Countries

Interview Guides & Observation Checklist


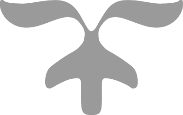


Meesha iqbal

november 28 2023

Contents

[Observation Checklist 2](#_Toc156132383)

[In-depth Interview Guideline for Policymakers 7](#_Toc156132384)

[In-depth Interview Guideline for Hospital Managers & Service Providers 10](#_Toc156132385)

[In-depth Interview Guide – Community Leaders 13](#_Toc156132386)

# Observation Checklist

| Observation Checklist: This checklist will be completed by PI of the study at every healthcare facility. It accounts for all domains of HSR expect "Choice of Provider," which cannot be observed and will be assessed only via interviews |  |
| --- | --- |
|  | Rating |
| Respect for Dignity (Assess the role of the doctor in this regard by 1 to 5 where 1-completely unsatisfactory and 5-completely satisfactory.) |  |
| (Greeting the patient) Patients expect that the doctor will greet and welcome the patient, make the patient feel comfort, accept the patient cordially, reply the patient's greetings and ask the patient's well being. | 1, 2, 3, 4, 5, NA |
| (Privacy during treatment) Patients should be treated and examined in a manner that respects their privacy. Privacy of the body is defined as preventing undue exposure of the body. Privacy includes examining the patient separately. If a male doctor is examining a female patient, a female nurse/ service provider should be present during examination. | 1, 2, 3, 4, 5, NA |
|  |  |
| Respect for Autonomy (Assess the role of the doctor in this regard by 1 to 5 where 1-completely unsatisfactory and 5-completely satisfactory. ) |  |
| (Right to information) Patients expect the doctor would explain everything to them, such as cause of the disease, diagnosis (at least the name of the disease), prognosis and severity, treatment (at least explaining prescription), other treatemrnt options, the side effects of the drugs (if any), the result of diagnostic tests (if any), preventive measures of disease (Diet) etc. | 1, 2, 3, 4, 5, NA |
| (Right to be consulted about treatment) Patients expect that the doctor would explain about the treatment of their diseases like which medicines have been given and why, how to take those medicines etc. In case of surgical requirement, the doctor should explain the details of surgery, its adverse effects, risks involved, time to recovery etc. The doctor should also explain alternate medical treatment options and their consequences if the patients do not want to opt for surgery. | 1, 2, 3, 4, 5, NA |
| (Informed consent) Observe the process of taking consent from the patients specially for minor procedures such as inserting a canula, nasogastric tube, urinary catheter, vacciination for children etc. The service providers should explain the process of the procedure to the patients, its possible side effects and risk involved. The patients should understand the process before permitting the providers to proceed. | 1, 2, 3, 4, 5, NA |
| (Informed consent - record review) - Review the "informed consent" forms in the surgical department. Read them for offering complete information and rate them on a scale of 1 to |  |
| (Right to refuse treatment) A good practice is that, the patient should participate in making decisions regarding treatment. He should be informed about different treatment options, treatment cost and advantages and disadvantages of each option. The doctor should help the patient to choose the best option by considering patient's personal and social conditions and the doctor should respect the patient's choice and the patient should have the right to refuse treatment if for any reason he/ she does not consider it appropriate. | 1, 2, 3, 4, 5, NA |
|  |  |
| Respect for Confidentiality |  |
| Patients expect that their information will be kept confidential and all measures will be taken to protect the information from reaching anyone. Observe how the reports and documents of patients are kept in the wards and assess confidentiality on a scale of 1 to 5 with 1 being unsatisfactory and 5 being satisfactory. | 1, 2, 3, 4, 5, NA |
|  |  |
| Quality of Basic Amenities |  |
| (Clean surroundings) The healthcare facilities should be clean. Regular maintenance of facilities is essential. Assess the facility in terms of cleanliness on a scale of 1 to 5 with 1 being unsatisfactory and 5 being satisfactory. | 1, 2, 3, 4, 5, NA |
| (Sufficient ventilation) Patients expect that the healthcare facilities should be well aerated and not stuffy. Assess the facility in terms of ventilation on a scale of 1 to 5 with 1 being unsatisfactory and 5 being satisfactory. | 1, 2, 3, 4, 5, NA |
| (Adequate seating) Patients expect that they should have sufficient seating to wait at the healthcare facilities. They should also have proper seating during consultation with the healthcare providers. Assess the facility in terms of seating and furniture on a scale of 1 to 5 with 1 being unsatisfactory and 5 being satisfactory. | 1, 2, 3, 4, 5, NA |
| (Food options) The healthcare facilities should offer food options for patients. This is specifically important for facilities offering in-patient care including secondary and tertiary care facilities. Assess the quality of food offered to admitted patients on a scale of 1 to 5 with 1 being unsatisfactory and 5 being satisfactory. | 1, 2, 3, 4, 5, NA |
| Assess the quality and variety of food available for purchase in the healthcare facility (e.g. cafeteria/ restaurants etc.) on a scale of 1 to 5 with 1 being unsatisfactory and 5 being satisfactory. | 1, 2, 3, 4, 5, NA |
| (Clean rest rooms) Patients expect that there should be clean rest rooms for their use in healthcare facilities. Assess the facility in terms of rest rooms on a scale of 1 to 5 with 1 being unsatisfactory and 5 being satisfactory. | 1, 2, 3, 4, 5, NA |
| (Drinkable water) Patients expect that there should be drinkable water available at the healthcare facilities. It could be in the form of water stations, coolers, bottled water etc. Assess the facility in terms of drinkable water on a scale of 1 to 5 with 1 being unsatisfactory and 5 being satisfactory. | 1, 2, 3, 4, 5, NA |
| (Clean linen) Patients expect that clean linen should be provided to them if admitted in the healthcare facility. This also includes provision of blankets as per need. Assess the facility in terms of availability of clean linen on a scale of 1 to 5 with 1 being unsatisfactory and 5 being satisfactory. | 1, 2, 3, 4, 5, NA |
| (Appropriate temperature) Patients expect that the internal temperature of the healthcare facility should be bearable. It should not be too cold in Winters and too hot in Summers. Assess the facility in terms of temperature control on a scale of 1 to 5 with 1 being unsatisfactory and 5 being satisfactory. | 1, 2, 3, 4, 5, NA |
|  |  |
| Access to Social Support Networks during Care |  |
| (Visits by friends and relatives) Patients expect that their friends and families will be allowed to visit them if they are admitted. There should be well-defined visiting hours and visitors should not be allowed to distrurb the patients other than the fixed timings. Assess the facility in terms of allowing visitors to visit on a scale of 1 to 5 with 1 being unsatisfactory and 5 being satisfactory. | 1, 2, 3, 4, 5, NA |
| (Connection with outside world: TV newspaper, radio, internet etc.) Patients expect that if they are admitted to the healthcare facility, they should be allowed to maintain contact with the outside world by means of newspaper, television, radio, internet etc. Assess the facility in terms of provision of such sources on a scale of 1 to 5 with 1 being unsatisfactory and 5 being satisfactory. | 1, 2, 3, 4, 5, NA |
| (Religious practices) Patients expect that they should be allowed to practice theor religious norms if they admitted to the healthcare facility. It should be noted that these should not disturb the comfort of other patients. Assess the facility in terms of allowing to carry out religious practices (such as offering prayers, reciting quran etc.) on a scale of 1 to 5 with 1 being unsatisfactory and 5 being satisfactory. | 1, 2, 3, 4, 5, NA |
| (Support to family of patients) Patients expect that their families should be given support if they are admitted to the hospital. Foe example if a woman is admitted to the labor room for child-birth, one attendant should be allowed to stay with the patients. The attendant should be given reasonable space to stay with the patient (such as a couch/ bed etc.). Assess the facility in terms of giving support to the family of patients on a scale of 1 to 5 with 1 being satisfactory and 5 being unsatisfactory. | 1, 2, 3, 4, 5, NA |
|  |  |
| Prompt access to care |  |
| (Waiting time) Patients expect that the waiting time should be reasonable for emergency as we as well non-emergency procedures. Assess the waiting time tof the healthcare facility | 1, 2, 3, 4, 5 |
|  |  |
| Attention and Clarity of Communication (Assess the role of the doctor in this regard by 1 to 5 where 1-completely unsatisfactory and 5-completely satisfactory.) |  |
| (Evoking understanding) It is extremely important that the patient understands all suggestions or explanations given by the doctor. The doctor should be sure that the patient understands him. Sometimes the doctors ask patients about their understanding of causes, diagnosis, prognosis, treatment etc. | 1, 2, 3, 4, 5, NA |
| (Enough time) Patients expect that the doctor would give them the opportunity to ask questions and also give appropriate answers to the patients. | 1, 2, 3, 4, 5, NA |
| (Use of jargon) One of the most important impediments for patients to understand doctor's advice is the medical terminology or Jargon), professional language etc. So, the doctor should avoid such language or explain it if used. Some examples of jargon include "pus cells," "nebulize" etc. | 1, 2, 3, 4, 5, NA |
| (Insightful listening) Patients expect that the doctor not only listen to their disease with patience and attention, but also listen to their questions with patience and attention. Some of the behaviors of the doctors that might express attention and patience might be shaking head while listening, looking at the patient, asking question to hear more, the nuances of voice, smiling, some interest revealing words (e.g.,: Well, hmm, etc.) and so on. | 1, 2, 3, 4, 5, NA |
| (Interruptions during consultations) Patients do not expect any sort of disturbance (interruption) during consultation. Some examples of interruptions include making or taking a call over the telephone, entrance of medical representatives, calling someone else inside the room for purposes other than the welfare of the patient etc. | 1, 2, 3, 4, 5, NA |
|  |  |
| Guidance (Assess the role of the doctor in this regard by 1 to 5 where 1-completely unsatisfactory and 5-completely satisfactory.) |  |
| (Healthy lifestyle & disease prevention) Patient expects that the doctor along with the treatment of the disease would also explain in details about diet, which foods are allowed and which are forbidden, prevention of the disease for which he has gone to the doctor, how to remain away from it etc. as well as lifestyle modification, preventive advice etc. Some examples include how to escape from the diarrhea (hand washing, use of sanitary latrines, etc.); protecting the child from catching cold who is suffering from pneumonia; advising the patient suffering from venereal diseases to use condom; maintaining cleanliness and drinking more water in case of UTI; eating less spicy food, drinking more water in case of PUD's; avoiding sweet foods in case of Diabetic patients; avoiding oily food, weight loss, eating less, taking precautions for preventing common fever and cold (wearing warm clothes, drinking warm water) in case of cardiac diseases and so on. | 1, 2, 3, 4, 5, NA |
| (Follow-up) Patients expect that the doctors would facilitate post treatment follow-up and give them a follow-up plan. Complete follow-up plan could be: When the patient would meet the doctor again; in which case the patient should contact the doctor before; if necessary, how the patient can reach the doctor; providing a contact e number to the patient; telling about follow-up costs; follow-up should be at free of cost; to write down what the patient should come up with at the time of follow-up (or at least tell); telling to inform the doctor immediately if any of the side effects of treatment arise etc. | 1, 2, 3, 4, 5, NA |
| (Access to medicines and diagnostic services) Patients generally do not know from where to access medicines and diagnostic facilities. This is specially true for primary care facilities, where people have to travel to other places to access medicines and diagnostic services. The doctors should guide the patients about the available options for running diagnostics and purchasing medicines. | 1, 2, 3, 4, 5, NA |
|  |  |
| Financial Sensitivity (Assess the role of the doctor in this regard by 1 to 5 where 1-completely unsatisfactory and 5-completely satisfactory. ) |  |
| (Understanding SES) Patients expect that the doctor would try to understand the socio-economic condition of the patient before providing treatment. Example of trying to understand the financial condition of the patient may be: Asking the patient directly about his income or whether he would be able to bear the treatment cost; Asking him indirectly (such as, asking his profession); if the patient tells. | 1, 2, 3, 4, 5, NA |
| (SES and Treatment Cost) Patients expect that the doctors would give them idea about treatment cost before starting treatment. Example of giving idea about cost of treatment may be: How much would be needed to complete the treatment; how long the treatment may continue; what impact the patient would be able to put on his ability of income during and after receiving treatment. | 1, 2, 3, 4, 5, NA |
| (Financial assistance) If the patients cannot afford treatment and management of their disease, they expect some financial assistance from the healthcare facility. Some examples for doctors could be prescribing low cost antibiotics; taking less or no consultation fee (in case of private doctors); helping patients from 'poor fund'; helping getting free medicines from the hospital (in case of government doctors);giving time and advice to collect money; focusing on the history and physical examination to avoid investigation; prescribing the essential tests only; cutting the commission paid to the doctor for each test; recommending that treatment method to the patient which saves money (meeting the nutritional needs from domestic sources, suggesting the pregnant woman to spend money for nutritious food instead of repeated ultra sonography etc.) and so on.  Additionally, some hospitals also have donations, funds, philanthropy societies that help such patients. The service providers should inform and refer the patients to such avenues if need be. | 1, 2, 3, 4, 5, NA |
|  |  |
| Coordination & Continuity of Care (Assess the role of the doctor in this regard by 1 to 5 where 1-completely unsatisfactory and 5-completely satisfactory. ) |  |
| (Communication among providers) Patients expect that the doctor would discuss with his colleague, another nearest doctor or anybody having knowledge about the disease if the doctor has some confusion or does not understand anything clearly (such as inability to understand X-ray, ECG and ultra sonogram, inability to diagnose skin diseases confidently etc.), about the treatment. Some cases also require a team of experts for proper management. | 1, 2, 3, 4, 5, NA |
| (Referral services) Patients expect that the doctor would refer the patient to another doctor immediately, if he cannot diagnose or treat the disease himself or it is out of the scope of the healthcare facility. The doctor should properly guide the patient by telling where to go, whom to go to, writing or explaining the address of the hospital and refer the patient with care. | 1, 2, 3, 4, 5, NA |
|  |  |
| Building trust (Assess the role of the doctor in this regard by 1 to 5 where 1-completely unsatisfactory and 5-completely satisfactory. ) |  |
| (Illegal activities) Patients expect service-oriented attitude from the doctor and consider business-oriented attitude as unwanted. Behaviors expressing business oriented attitude may be: telling the patient to do test from any specific diagnostic center, encouraging to buy medicines ofa specific pharmaceutical company, taking money from patients forcibly, taking money from pharmaceutical companies, taking money from patients against free services, telling the patient under consultation of a govt. doctor to go to a private clinic, etc. Examples of service-oriented attitudes may be: asking the patient's ability to bear the cost of treatment, if necessary assisting the patient in getting low-cost medical care and so on. | 1, 2, 3, 4, 5, NA |
| (Accountability mechanisms) Assess the healthcare facility in terms of accountability mechanisms for clinical care. Examples of accountability mechnisms include feedback given via reviews etc.) | 1, 2, 3, 4, 5, NA |

# In-depth Interview Guideline for Policymakers

| Date of Interview: | Start Time: | | End Time: |
| --- | --- | --- | --- |
| Interviewer’s Name: | | Location | |
| Interviewee’s Name: | |  | |
| Age: | Gender: Male / Female | | Occupation: |
| Education: | Religion: | |  |
| Organization of the Interviewee |  | |  |

**Note to IDI Facilitator**

Before beginning the interview, make sure the client has provided written informed consent. Thank her/ him for agreeing to participate. Introduce yourself and study. Provide additional information if requested by the respondent. Take the interviews in a private setting; avoid interviewing in the presence of another person, as these may bias the responses. Audio-record the interview after seeking permission from the participant.

**Semi-structured Interview Guide**

Q 1: Tell me about yourself, your work, education, etc.

Q 2: Tell me about your experiences of healthcare being provided in tertiary care hospitals of *(country name).*

Probes:

- What are your views about non-medical care being provided in tertiary care hospitals?

Q 3: Is there a difference in the healthcare being provided in public vs private sector hospitals?

Probes:

- How does non-medical part of treatment differ across the sectors e.g. treating patients with respect, respecting their autonomy, giving them the choice of provider etc.

Q 4: How is **privacy** of patients taken care off in the hospitals?

- Public vs. private
- How important is it to maintain their privacy?

Q 5: What are your views and experiences of **autonomy** being provided to patients for dealing with their own ailment?

- Public vs. private
- How important is it?

Q 6: What is the situation of explaining to the patients their disease, diagnosis, treatment options, side effects, prognosis, medication, etc.? Please explain your response.

Probes:

- What are the constraints of doing so?
- Public vs. private
- How essential is it to allow the patients to ask questions?

Q 7: What is the situation of maintaining **confidentiality** of patients information specially in regards to tertiary care hospitals?

Q 8: How quickly do patients get care in tertiary care hospitals?

Probes:

- What are the bottlenecks in providing timely care?
- Public vs. private?
- How do patients suffer due to this?

Q 9: What is the quality of **basic amenities** like in the tertiary care hospitals? *(for each question, ask private vs. public)*

Probes:

- Is clean water available to the patients and their attendees?
- Is healthy food available to patients requiring hospital admission?
- What is the state of the waiting rooms, rooms and toilets?
- What is the state of proper ventilation and furniture in the hospitals?

Q 10: What is the state of **clarity of communication** between the healthcare providers and patients?

Probes:

- Considering that the majority of the population is not literate, due to think there are barriers in communication between the service providers and patients?
- How does this impact management of diseases and ailments?
- What are the other bottlenecks in ensuring clarity in communication?

Q 11: Is a common man given the **choice of provider** when he goes to a tertiary care for consultation? If not, why not?

Probes:

- Is this not important?
- Public vs. private?
- Does this affect the management of patients in any way?

Q 12: What **social support networks** are available to patients of tertiary care hospitals e.g. Parkinsonism clubs, Alzheimer’s club, Ante natal clubs etc.

Probes:

- What is the social support mechanism of patients, apart from these formal clubs?
- Are patients’ relatives and friends allowed to care for them during their hospital stays?
- Are religious practices allowed in the hospital, for all patients?

Q 13: What are the overall challenges in improving responsiveness of healthcare in tertiary care hospitals?

Q 14: What solutions do you think can be applicable in this context?

Q 15: Would you like to add anything else?

# In-depth Interview Guideline for Hospital Managers & Service Providers

| Date of Interview: | Start Time: | | End Time: |
| --- | --- | --- | --- |
| Interviewer’s Name: | | Location/ Hospital: | |
| Age: | Gender: Male / Female | | Occupation: |
| Education: | Religion: | |  |
| Role in the Hospital: |  | |  |

**Note to IDI Facilitator**

Before beginning the interview, make sure that the interviewee has provided written informed consent. Thank her/ him for agreeing to participate. Introduce yourself and study. Provide additional information if requested by the respondent. Take the interviews in a private setting; avoid interviewing in the presence of another person, as these may bias the responses. Audio-record the interview after seeking permission from the participant.

**Semi-structured Interview Guide**

Q 1: Tell me about yourself, your education, medical life, career, family.

Q 2: Why did you choose to come to the medical profession?

Q 3: How is your experience about working in this profession?

Q 4: Could you describe your consultation process in detail? For example, history taking, general physical examination, systemic examination, consultation with colleagues, prescription for lab diagnosis, etc.). Do you greet the patient in any way?

Q 5: Do you think it is necessary for a physician to be responsive, or do you think only clinical competency is enough? How do you compare these? [By responsiveness I mean “the social actions that health service providers do to meet the ‘legitimate expectations’ of service seekers”. For example, when a patient comes to you she/he may expect that you would allow her/him to ask questions regarding disease/ health condition; or she/he may expect that you would maintain privacy while examining her/him, etc.]

Q 6: What according to you should be the non-medical expectations of the patients? What things a patient may expect from you, which are not directly related to her/his therapy?

Probe:

- Many of these expectations may be considered legitimate expectations; but are there any such expectations from patients that you don’t consider legitimate? Please explain.

Q 7: In an ideal situation, what would you add to your consultation process, which you do not perform presently?

Probes:

- We all want to provide services to patients with responsiveness; but this is not often possible due to certain barriers?
- What according to you are those constraints (e.g., insufficient number of HRH, unavailability of infrastructure, unavailability of equipment, lack of administrative support, anything else?

Q 8: Is there a difference in service provision between public sector, private sector, and informal sector?

Q 9: Do you think asking for consent of the patient before performing any procedure is necessary? Why do you think so? If you do not take consent, what are the constraints?

Q 10: Tell me about your views regarding the privacy of the patients?

Probes:

- Do the patients ever ask for it?
- What do you do if they ask?
- In what type of situations do you maintain it?
- Where do you think it is not necessary?
- Is it possible for you to maintain it in every instance that you think it is important?
- What are the constraints in maintaining it?

Q 11: Do you think it is important to explain to the patients their disease, diagnosis, treatment options, side effects, prognosis, medication, etc.? Please explain your response.

Probes:

- What are the constraints of doing so?

Q 12: How essential is it to allow the patients to ask questions?

Probes:

- How do you feel when someone asks questions?
- To what extent do you feel comfortable in answering their questions?
- What types of questions do you expect from the patients?
- Do you (or your colleagues) ever get annoyed with the patients’ questions?
- Why, or in what situations?

Q 13: How does the physician react to a patient if she/he wants to consult a different physician?

Probes:

- Do you think it is alright, or not, for a patient to refuse to consult one physician and want to consult a different physician?
- Do you think patients have right to consult the physicians of their choice (male/female or other considerations)?

Q 14: When do you (or physicians in general) refer a patient to a different doctor? Do you consult with other physicians in conditions that you are not comfortable in treating?

Q 15: What is your opinion regarding respecting the patients?

Probes:

- Do you think the physicians respect patients? How do you think respect towards the patient is commonly breached?

Q 16: Are patients concerned about maintaining the confidentiality of patient information?

Probes:

- Is there any way to maintain the confidentiality of the patients?
- What happens if you are asked by some other person or organization (drug company, hospital, insurance company, research organization, etc.) to reveal patient information?

Q 17: Do you ever inform the patients about the preventive aspects of the disease? Or do you think it is beyond your responsibility? Please share your views about this.

Q 18: How much time do you give per patient in general? How much time would be ideal?

Q 19: Do you display empathy to the patients regarding their ailments? How do you do this? Is it important?

Q 20: How easily/ frequently are providers misunderstood by patients? Why and how?

Q 21: Is there any way of engaging the patients or their family members in the care process? Do you do this? Why, why not?

Q 22: Is there any way to maintain the continuity of care by the same physician (i.e.,you)? How do you follow-up your patients?

Q 23: Do you consider the SES of the patient before prescribing drugs or diagnostic tests?

Q 24: What are your views regarding gender sensitivity, cultural sensitivity etc.? Is it important to talk to the patient in a familiar language?

Q 25: Is there anything else that you want to add?

# In-depth Interview Guide – Community Leaders

| Date of Interview: | Start Time: | | End Time: |
| --- | --- | --- | --- |
| Interviewer’s Name: | | Location: | |
| Age: | Gender: Male / Female | | Occupation: |
| Education: | Religion: | |  |

**Note to in-depth interview (IDI) Facilitator**

Before beginning the interview, make sure the client has provided written informed consent. Thank her/ him for agreeing to participate. Introduce yourself and study. Provide additional information if requested by the respondent. Take the interviews in a private setting; avoid interviewing in the presence of another person, as these may bias the responses. Audio-record the interview after seeking permission from the participant.

**Semi-structured Interview Guide**

Q1: Tell me about yourself, your family, your work, education, where you are from, etc.

Q 2: Tell me about the people of this community? (ask about their socio-demographic characteristics)

Q3: Where do the people of this community generally go to seek healthcare? (Probes: what are specific public healthcare facilities where people go or not go to seek healthcare)

Q 3: What types of service providers are available within the reach of this community (distance, cost, etc.)? How do you decide which provider to consult (public, private, informal, any other)? Which providers do you consult for what types of conditions?

Q 4: What entitlements do you think you have in seeking services from a service provider?

Q 5: Apart from the technical care provided (diagnosis, advice, treatment), what other expectations from the healthcare facilities do people have, e.g., being treated with respect, privacy, confidentiality, etc.? What are your expectations apart from treatment? Are these expectations met in the consultations? How? Why, why not?

Q 6: How do you find the environment of the healthcare facilities available in the community?

- Do you think there is adequate space allotted to patients staying over night? How many patients have to share one room? How many should share the room?
- What are some of the problems faced by patients while staying overnight in the hospital?
- What is the condition of the toilets/ rooms/ waiting rooms/ cafeteria?
- What sort of food is provided in the hospital? What is the quality of food? What is your perception about the diversity of food provided?
- Are friends, family and visitors allowed to visit the patients in the hospital? Do they have to face any hurdles or issues in doing so? If yes, what are these?

Q 7: Apart from clinical care, what are the specific areas that should be improved in providing care to the people? (Probe for suggestions for improvement)

Q 8: Do you want to add anything else?
