# Supplementary material for: Rethinking Health Systems Responsiveness in Low- and Middle-Income Countries: Validation Study
Source: JMIR Res Protoc. 2024 Sep 18;13:e59836. doi: 10.2196/59836 (PMC11447431; doi:10.2196/59836)
Supplement: Multimedia Appendix 4 [file resprot_v13i1e59836_app4.docx]

#### **RETHINKING HEALTH SYSTEMS RESPONSIVENESS IN LOW-AND MIDDLE-INCOME COUNTRIES**

Sample Size Calculation, Distribution and Sampling Strategy

#### Sample Size Calculations

Sample size has been calculated separately for primary, secondary and tertiary care hospitals. Secondary and tertiary care hospitals entertain inpatients as well as outpatients, whereas the primary care facilities generally offer outpatient services only (although RHCs theoretically have 10 to 20 beds). Thus, we have calculated sample size for inpatients as well as outpatients for secondary and tertiary care facilities, and only for outpatients for primary care facilities. For both groups of patients, sample size was independently calculated for each domain of responsiveness (Eight existing domains, Table 1). The anticipated frequencies were taken from World Health Survey, done in Pakistan in 2003-2004, as no latest estimates are available.^[21]^ As the sample would be drawn from healthcare facilities (clusters), it is important to adjust the design effect. An effort was made by the research team to find Intra-class correlation (ICC) to calculate the design effect (D.E = 1 + [cluster size – ICC]). However, ICCs were available at community level for Pakistan,^[45]^ but have not been calculated for healthcare facilities. Thus, a ***design effect of 1.5***,^[46, 47]^ which is usually taken in national surveys in Pakistan, was adopted for the study. For both groups of patients, domain of “dignity” had the highest sample size: n=226 for out-patients and n=225 for in-patients. Thus, these numbers were selected as follows:

- Tertiary healthcare facilities: n= 226 out-patients, and n= 225 inpatients (N=451)
- Secondary healthcare facilities: n= 226 out-patients, and n= 225 inpatients (N=451)
- Primary healthcare facilities: n= 226 out-patients
- Total sample size of the study = 1,128 participants

| **Table 1: Sample size assumptions and calculations for outpatients from primary, secondary and tertiary care facilities** | | | | | | |
| --- | --- | --- | --- | --- | --- | --- |
| **Final Sample Size** | **Design Effect** | **Sample size (n)** | **Confidence Level** | **Absolute Precision** | **Proportions (%)**  **(Anticipated frequency)** | **Domains of responsiveness** |
| 226 | 1.5 | 151 | 95% | 8% | 50.4 | Dignity |
| 218 | 1.5 | 145 | 95% | 8% | 59.3 | Autonomy |
| 225 | 1.5 | 150 | 95% | 8% | 46.8 | Confidentiality |
| 199 | 1.5 | 133 | 95% | 8% | 67.2 | Prompt attention |
| 219 | 1.5 | 146 | 95% | 8% | 58.5 | Basic Amenities |
| 220 | 1.5 | 147 | 95% | 8% | 57.7 | Choice of provider |
| 225 | 1.5 | 150 | 95% | 8% | 52.9 | Communication |
| A sample of 226 out-patients will be drawn from primary, secondary as well as tertiary healthcare facilities. Total outpatients = 226 *3 = 678 participants | | | | | | |
| **Sample size assumptions and calculations for in-patients from secondary and tertiary care facilities** | | | | | | |
| **Final Sample Size** | **Design Effect** | **Sample size (n)** | **Confidence Level** | **Absolute Precision** | **Proportions (%)**  **(Anticipated frequency)** | **Domains of responsiveness** |
| 225 | 1.5 | 150 | 95% | 8% | 53.0 | Dignity |
| 215 | 1.5 | 143 | 95% | 8% | 61.0 | Autonomy |
| 223 | 1.5 | 149 | 95% | 8% | 55.2 | Confidentiality |
| 206 | 1.5 | 137 | 95% | 8% | 64.8 | Prompt attention |
| 218 | 1.5 | 145 | 95% | 8% | 59.2 | Basic Amenities |
| 217 | 1.5 | 145 | 95% | 8% | 59.6 | Choice of provider |
| 223 | 1.5 | 149 | 95% | 8% | 55 | Communication |
| 222 | 1.5 | 148 | 95% | 8% | 56.7 | Access to social support |
| A sample of 225 in-patients will be drawn from secondary as well as tertiary healthcare facilities. Total inpatients = 225 *2 = 450 participants | | | | | | |
| Total sample size (in-patients + outpatients) = 678 + 450 = 1,128 | | | | | | |

#### Sampling Strategy and Sample Distribution across Tertiary Healthcare Facilities

There are a total of six tertiary healthcare facilities in district Rawalpindi. Three provide comprehensive healthcare services (Rawalpindi Teaching Hospital, Holy Family Hospital, and Benazir Bhutto Hospital, Rawalpindi, Rawalpindi), and the other three provide specialized care (Rawalpindi Institute of Urology and Transplantation, Rawalpindi Institute of Cardiology, Syed Muhammad Hussain Govt. T.B Sanatorium Samli). The three tertiary healthcare facilities which provide comprehensive healthcare were selected for the study. The sample will be drawn from these three hospitals, from inpatients and outpatients across the departments of Medicine, Surgery, Gynae/ Obs and Pediatrics. Probability proportionate to size sampling technique has been applied to draw the sample from three tertiary care hospitals, according to their bed-capacity as shown in Table 2. Within each healthcare facility, an equal number of participants will be drawn from in-patient and outpatient departments. Furthermore, the sample will be equally drawn from Medicine, Surgery, Gynae/ Obs and Pediatrics departments.

Table 2: Distribution of sample across tertiary and secondary healthcare facilities

| **Tertiary healthcare facilities (n=3)** | | | | |
| --- | --- | --- | --- | --- |
| **Tertiary healthcare facility** | **Bed Capacity** | **Probability for each hospital** | | **Sample size for each hospital (rounded off to next whole number)** |
| Rawalpindi Teaching Hospital | 510 | 0.216102 | | 98 |
| Holy Family Hospital | 1100 | 0.466102 | | 211 |
| Benazir Bhutto Hospital | 750 | 0.317797 | | 144 |
| **Total sample size 453** | | | | |
| **Secondary healthcare facilities (n=3)** | | | | |
| **Healthcare facility** | **Bed Capacity** | **Probability for each hospital** | **Sample size for each hospital (rounded off to next whole number)** | |
| Tehsil Headquarter Hospital, Taxila | 100 | 0.454545 | 205 | |
| Tehsil Headquarter Hospital, Kahuta | 60 | 0.272727 | 123 | |
| Tehsil Headquarter Hospital GujarKhan | 60 | 0.272727 | 123 | |
| **Total sample size 451** | | | | |

#### Sampling Strategy and Sample Distribution for Secondary Healthcare Facilities

There are a total of seven secondary healthcare facilities in district Rawalpindi. Three facilities were selected based on feasibility: Tehsil Headquarter Hospital, Taxila; Tehsil Headquarter Hospital, Kahuta; Tehsil Headquarter Hospital, Gujar Khan.

The sample will be drawn from these three hospitals, from inpatients and outpatients across the departments of Medicine, Surgery, Gynae/ Obs and Pediatrics. Probability proportionate to size sampling technique has been applied to draw the sample from three facilities according to their bed-capacity as shown in Table 8. Within each healthcare facility, an equal number of participants will be drawn from in-patient and outpatient departments. Furthermore, the sample will be equally drawn from Medicine, Surgery, Gynae/ Obs and Pediatrics departments.

#### Sampling Strategy & Sample Distribution for Primary Healthcare Facilities

The primary healthcare facilities of district Rawalpindi include 91 dispensaries, 99 BHUs and 9 RHCs. It has been decided by research team to draw sample from BHUs and RHCs. Dispensaries will not be included in the study as these are generally used for dispensing medications on prescription of doctors and do not represent patient-provider interface. Three RHCs and three BHUs will be included in the study based on convenience; to draw a sample of 226 patients (from the out-patient department). The sample has been equally distributed across the facilities (Table 3). Although distinct departments are not a part of primary healthcare facilities, the data collectors will make an effort to collect data from medical, surgical, gynae/obs and pediatrics patients, equally from each facility. If segregation is not possible, data will be collected from any patient coming to the facility.

Table 3: Distribution of sample across primary healthcare facilities (BHUs and RHCs)

|  | Sample size |
| --- | --- |
| Basic Health Units (n=3) | |
| Basic Health Unit, Brehma, Taxila, Rawalpindi | 38 |
| Basic Health Unit, Bishandot, Kallar Syedan, Rawalpindi | 38 |
| Basic Health Unit, Matore, Kahuta, Rawalpindi | 38 |
| Rural Health Units (n=3) | |
| Rural Health Center, Mandra, Gujar Khan, Rawalpindi | 38 |
| Rural Health Center, Thoha Khalsa, Kahuta, Rawalpindi | 38 |
| Rural Health Center, Khayaban-i-Sir Syed, Rawalpindi | 38 |

Figure 1 shows the sample distribution of the study.

Figure 1: Sampling distribution across primary, secondary and tertiary healthcare facilities (n=12).
